# Platforms, risk perceptions, and reporting: the impact of illicit drug advertisements on social media among UK secondary students

**DOI:** 10.1186/s12954-025-01299-5

**Published:** 2025-10-03

**Authors:** Ashly Fuller, Marie Vasek, Enrico Mariconti, Shane D. Johnson

**Affiliations:** 1 Dawes Centre for Future Crime, University College London, London, UK; 2 Department of Security and Crime Science, University College London, London, UK; 3 Department of Computer Science, University College London, London, UK

**Keywords:** Social media, Illicit drugs, Survey, Young people, Drug online safety

## Abstract

**Background:**

The sale and advertisement of illicit drugs on social media is a rapidly evolving landscape. While existing research has focused on market structures, purchase strategies, and platform types, there is limited understanding of how viewing such content affects young people. This study aims to examine young people’s experiences with illicit drug ads on social media and explore the relationship between exposure to these ads and their attitudes and behaviours towards drug use.

**Methods:**

We conducted an online survey of students aged 13–18 (N = 1,151), distributed to UK schools by two drug education charities. Participants had a mean age of 14.7 years (SD = 1.28), and gender distribution was roughly equal (51% female, 47% male).

**Results:**

Most participants encountered drug-related content on social media, with 29% having seen illicit drugs advertised for sale without actively searching for them. While Snapchat, Instagram, and TikTok were the most common platforms for these ads, rates of exposure per unit of time were found to vary across platforms. Exposure to drug safety advice differed across platforms, with participants reporting encountering drug safety advice more frequently than illicit drug ads on TikTok for example, highlighting the potential for leveraging social media to promote drug safety. We also find significant associations between young people’s exposure to content and decreased risk perceptions, along with increased interest in and intention to buy illicit drugs.

**Conclusion:**

Our research is the first to provide a detailed understanding of platform exposure to illicit drug ads on social media, highlighting the need for research across diverse platforms. Despite our findings, the impact of exposure to drug ads remains unclear. We advocate for a new approach to studying this issue, integrating an online safety perspective.

**Supplementary Information:**

The online version contains supplementary material available at 10.1186/s12954-025-01299-5.

## Introduction

Five years after one of the first studies on this issue [1], the sale and advertisement of illicit drugs on social media has become a recognised phenomenon. As research in this area grows, regulatory efforts are gaining momentum. Landmark legislation, such as the UK’s Online Safety Act and the US’s Kids Online Safety Act [2, 3], have introduced specific responsibilities for service providers to address and prevent the sale of illicit drugs on their platforms, particularly to younger audiences. Industry initiatives include those led by Snapchat, Meta, and X which formed the *Alliance to Prevent Drug Harms* in July 2024 [4], in collaboration with the US State Department and the UN Office on Drugs and Crime (UNODC). Such initiatives further highlight the growing recognition of the problem and the increasing debate around who holds responsibility for addressing the expansion of drug markets on social media, whether that lies with platforms, governments, or both in tandem.

Online drug markets are highly adaptive, often exploiting unexpected platforms to advertise illegal products. While the most well-known platforms for illicit drug advertisements include Snapchat, Instagram, and Facebook [1, 5, 6], a BBC News [7] investigation recently uncovered nearly 3000 posts advertising the sale of Opioids on SoundCloud, a user-driven music streaming platform. Some of these remained online for over one year, with drug names and contact details embedded in the track titles. This reflects a growing trend highlighted in the literature [8]: platforms not traditionally seen as social media are increasingly being used to sell drugs. Theories of crime science suggest that where criminal opportunities exist, they are exploited [9–11]. Moreover, the Routine Activity Approach states that for a crime to occur a motivated offender must encounter a suitable target, absent a capable guardian [12]. Things that increase this convergence thus increase opportunities of crime. As young people increasingly maintain digital routines, advertising illicit drugs on social media platforms enables offenders to reach a receptive user base at scale. Furthermore, digital spaces that are not monitored by regulators are more likely to lack appropriate guardianship [12] and may become attractive targets for drug advertising. Recent evidence supports this, indicating that gaming platforms, clothing resale apps, dating apps, and even e-commerce websites are increasingly being used as key spaces for illegal drug advertisements [13–15].

The online sale of illicit drugs has evolved from darknet cryptomarkets to more accessible clear web platforms, driven by the ongoing need to balance convenience and anonymity for users [16–18]. With the rise of social media platforms characterised by user-friendly interfaces, forum-like structures, and opportunities for anonymity [19, 20], these spaces have naturally become new arenas for drug sales. This shift reflects how social media’s widespread adoption and design features facilitate easier and broader access to illicit substances. Alongside this shift, the way in which illicit drug advertisements are publicised and framed on social media is also changing. For instance, cannabis and body-enhancing drugs have been associated with ‘wellness’ and ‘healthy lifestyle’ content [21]. Prescription stimulants, or study drugs, are being presented within a culture of achievement and success, appearing alongside motivational quotes [22]. The growing sophistication in drug advertisements is further complicated by the legal grey area surrounding drug sales on social media: posts and accounts are becoming more professional, with sanitised, aesthetic images portraying illicit drugs as regular products.

Despite increasing regulatory and research attention, we may only be seeing the tip of the iceberg. Emerging drug types, innovative advertising strategies, and shifts in platform usage introduce new challenges and questions, particularly regarding their impact on young people and minors and the potential normalisation of viewing and purchasing illicit drugs on social media.

### Drug and social media use among UK youth: the potential impact of illicit drug advertisements

Drug use among young people in the UK has seen a general decline over the last 10 years [23]. For example, in NHS digital surveys, in 2023 13% of young people aged 11–16 reported ever taking drugs, which represents a reduction compared to 2021 (18%) and 2018 (24%) [24, 25]. Similarly, in 2023, 9% of pupils reported having taken drugs in the last year compared to 12% in 2021 [24, 25].

Apropos social media use, in 2022 the most popular social media and messaging platforms used by children aged 3–17 were YouTube, WhatsApp, TikTok, and Snapchat, all of which saw increased usage compared to 2021, unlike Facebook. Young people spent the most time on TikTok, averaging nearly two hours per day, followed by Snapchat at 1.5 h [26]. Reasons for going online were to play video games, communicate with others, and learn. Viewing content on video-sharing platforms was almost universal, but only 30% of 8 to 17-year-olds actively shared, commented, or posted, suggesting that most are passive consumers of online content [27].

When it comes to the positive and negative connotations of being online, most young people in the UK recognize the benefits of social media. According to [28], 73% of those aged 8–17 feel safe using these platforms, and 67% report feeling happy while using them—an 8% increase since 2021. However, this sense of safety coexists with significant risks: 29% of young people reported experiencing hurtful behaviour online, with this figure rising to 24% among 16 to 17-year-olds [27]. Similarly, 29% of 8 to 17-year-olds encountered worrying or harmful content, although this marks a decline from 36% in 2021. These statistics illustrate the dual reality of social media for young people: it is an integral part of their daily lives, offering connection and enjoyment, but it also exposes them to a variety of harms, that differ by gender and age. For instance, older teens and boys are more likely to view violent or inappropriate material [27, 29]. Cross-national evidence also shows a correlation with socioeconomic status (SES), with children from lower SES backgrounds being more likely to encounter harmful content online [30].

In summary, although drug use by young people in the UK is declining, young people are spending more time online while having to navigate harmful content and behaviours. In this context, it is important to ask what the implications of illicit drug advertisements on social media and messaging platforms are. While the presence of illicit drugs online may be unlikely to reverse the declining national trend in drug use, its increasing visibility on social media coupled with young users’ exposure to various types of harmful content, may contribute to a normalisation of illicit drug advertisements, and possibly use. The normalisation thesis in the sociology of drug use, which stems from the increasing observation of recreational drug use among young people, refers to the shift from viewing drug use as deviant and criminal to a growing societal acceptance of it [31–34]. Such changes have been argued to be driven by various factors including evolving social norms, greater drug availability, and increased use and knowledge of drugs [35, 36]. There appears to be a growing normalisation of drugs online, with the increasing presence of substances on social media and their portrayal becoming central to debates in drug studies, particularly around the potential impact of exposure on an individual’s own use [37], especially among teenagers [38].

### Why UK-based data are needed: the present research

Studies examining the strategies and motivations for users to advertise, sell and buy drugs on social media apps provide essential first-hand empirical data on this topic. These have done so by using digital ethnographies [5, 15, 22, 39–42] or a blend of interviews and surveys [1, 43–49]. While these studies have yielded important in-depth knowledge on the characteristics of users and the strategies used to buy or sell drugs, most of these studies are based outside the UK. In addition, their samples are purposive and relatively small, typically being between 30 and 1000 participants. One exception was van der Sanden et al. [50], whose study, based on a sample of 23,500 respondents aged 16 + from the New Zealand Drug Trends Survey (NZDTS), aimed to identify predictors social media use to purchase drugs. In the UK, the report *DM for Details* from the drug policy think tank *Volteface* [6] was the first to provide empirical data on young people’s experiences around drugs on social media, with the collecting data from 2000 survey responses, 24 interviews and 4 focus groups. However, the data were collected in 2019 and only from those aged 16–24 years. Since then, research considering younger adolescents has not been collected.

National surveys across the UK provide a comprehensive overview of young people’s trends around substance use. However, none examine the role of social media in relation to the purchase or consumption of illicit drugs. While the *NHS’s Smoking, Drinking and Drug Use among Young People in England* [24] and Scotland’s Scottish Schools Adolescent Lifestyle and Substance Use Survey (SALSUS) [51] collect useful data about where young people source illicit drugs from, the answer option for online sources is limited to ‘*the internet’* or ‘*website*’, which are unhelpful categories given existing knowledge of illicit drugs being sold online.

This study addresses these gaps by generating recent, UK-based data on adolescents’ experiences with drug-related content and activities on social media platforms.

### Research questions and hypotheses

With emerging platforms and evolving ways of presenting illicit drugs on social media, along with a lack of up-to-date empirical evidence on young people in the UK, it is crucial to gather data to accurately capture young people’s experiences with this phenomenon. The first question guiding this research is*: What are school students’ (13- to 18-year-olds) experiences around drugs and illicit drug advertisements on social media?* Given that young people are spending more time online and are likely to be increasingly exposed to such advertisements, it is also reasonable to suggest that this may contribute to the normalisation of illicit drug use. The second question this study aims to address is thus*: Does exposure to illicit drug ads influence young people’s attitudes and behaviours toward it?* Based on the literature and preliminary evidence, we outline two primary hypotheses related to the second research question. While more specific sub-hypotheses could be formulated, we focus here on the two most central ones:

#### H1

Exposure to illicit drug advertisements on social media is associated with a decreased perception of the risks associated with drug use.

#### H2

Exposure to illicit drug advertisements on social media is associated with an increased likelihood of purchasing drugs through social media platforms.

We acknowledge that H2 may appear self-evident at first glance, since exposure is arguably a prerequisite for purchasing drugs via social media. However, we view this as an empirical question rather than a given. Not all individuals exposed to drug-related content act on it, and understanding whether exposure translates into behaviour is particularly important in the context of illicit markets, where risk, trust, and access barriers may complicate this pathway. This hypothesis tests whether exposure is simply correlated with behaviour or whether it reflects a more systematic relationship that warrants attention from researchers and policymakers.

We conduct an online survey of UK students aged 13–18 to understand their exposure to, practices and attitudes around drugs being sold and advertised on social media. Researching young people under 18 is important as limited data exists for these age groups, who may be particularly vulnerable to the impacts of illicit drug ads. The aim of this study is to explore the relationship between exposure to drug advertisements on social media and young people’s attitudes and behaviours towards drug use.

While the term ‘social media’ is widely used, it remains difficult to define precisely. Scholars across disciplines have characterised it as a broad and evolving category, with overlapping and sometimes conflicting definitions based on features, user practices, or platform histories [52]. Terms such as ‘social networking sites’ or ‘micro-blogging’ are often used interchangeably with social media, yet distinctions remain: for example, between platforms with reciprocal ‘friending’ and those oriented around content broadcasting or algorithmic feeds [53, 54]. Others note that platforms once categorised as messaging apps now incorporate features commonly associated with social media, such as content sharing or group interaction [55]. Given these shifting boundaries, this paper adopts the broad definition proposed by [20]: *internet-based channels that enable users to interact and self-present, either in real time or asynchronously, to broad or narrow audiences (…) (p.50)*. This definition captures both traditional social media platforms (e.g. Instagram, TikTok) and messaging-based services with social affordances (e.g. WhatsApp, Telegram), which are central to understanding the contemporary digital drug trade.

## Methodology

### Survey design and procedure

The survey was co-designed with the *UK Drugs on Social Media* working group, which is chaired by the *Daniel Spargo-Mabbs Foundation*, and comprises UK stakeholders from the voluntary sector, law enforcement, government departments and social media companies. Co-design in health research has been shown to positively influence research [56, 57] and is being increasingly adopted in the field of digital health [58–60], including the development of digital mental health technologies for children and young people [61]. For example, co-design enables the integration of a range of views and experiences from stakeholders who are in direct contact with the population of interest. Moreover, the active participation of stakeholders in research projects has the potential to directly inform policy outcomes [62, 63] and enable the efficient diffusion of evidence across a variety of institutions and political settings [64–66].

The survey was conducted using Qualtrics XM and was composed of 35 questions (see supplementary material for a copy). It included questions about which social media platforms they used and how frequently, whether they had ever seen illicit drug ads, on which platforms and how often they saw them. In this survey, ‘illicit drugs’ were defined as ‘*any illicit/illegal drug, including controlled prescription drugs, such as Valium and Tramadol’*. This includes substances that are illegal to possess or supply in the UK (e.g. MDMA, cocaine), as well as prescription medications that are legal when prescribed but considered illicit when sold or promoted unlawfully online. Participants were asked to what extent they agreed with statements related to the risk of buying drugs on social media, how easy they believed they were to purchase, the quality of the drugs advertised and the likelihood of getting caught. Further questions explored reporting practices, personal views on whether they considered illicit drug content to be problematic, who they felt was responsible for dealing with such content, and whether they had also viewed harm reduction content.

Data were analysed descriptively to summarise sample characteristics and prevalence estimates, while ordinal logistic regressions were conducted to assess the influence of exposure to ads on risk perceptions, attitudes towards drug ads, drug purchasing behaviour, perceptions of responsibility, barriers to reporting, and whether respondents had ever reported illicit ads. All models included independent variables to control for the effects of age, gender and frequency of social media use, as these factors have been linked to adolescents’ access to drugs online [47, 48]. A country level (fixed effect) variable was also added to account for sample differences and variation in drug education policies, while school year was included to capture nuances that age alone may not reflect. Multicollinearity was assessed using the Variance Inflation Factor (VIF). Although age and school year slightly exceeded the recommended VIF threshold of 5, both were retained due to their distinct importance (models were separately run including and excluding each age and school year and showed that the results were comparable). However, for the sake of parsimony, only the estimated coefficients for the key predictor variable (whether respondents had seen drug advertisements on social media or not) are reported in Fig. 6 (See Appendices Tables 4 to Fig7 for the full models).

### Survey piloting

Pretesting, or piloting, is essential in survey research [67–70]. It ensures that questions are clear, options are sufficient, and that the survey flow is logical [71]. For surveys aimed at young people, these must align with their cognitive and linguistic skills [72, 73]. Pretesting minimizes misunderstandings that could affect the validity of the responses [74] and enhances survey quality, which can improve response rates and reduce missing data [75].

**Fig. 1:**
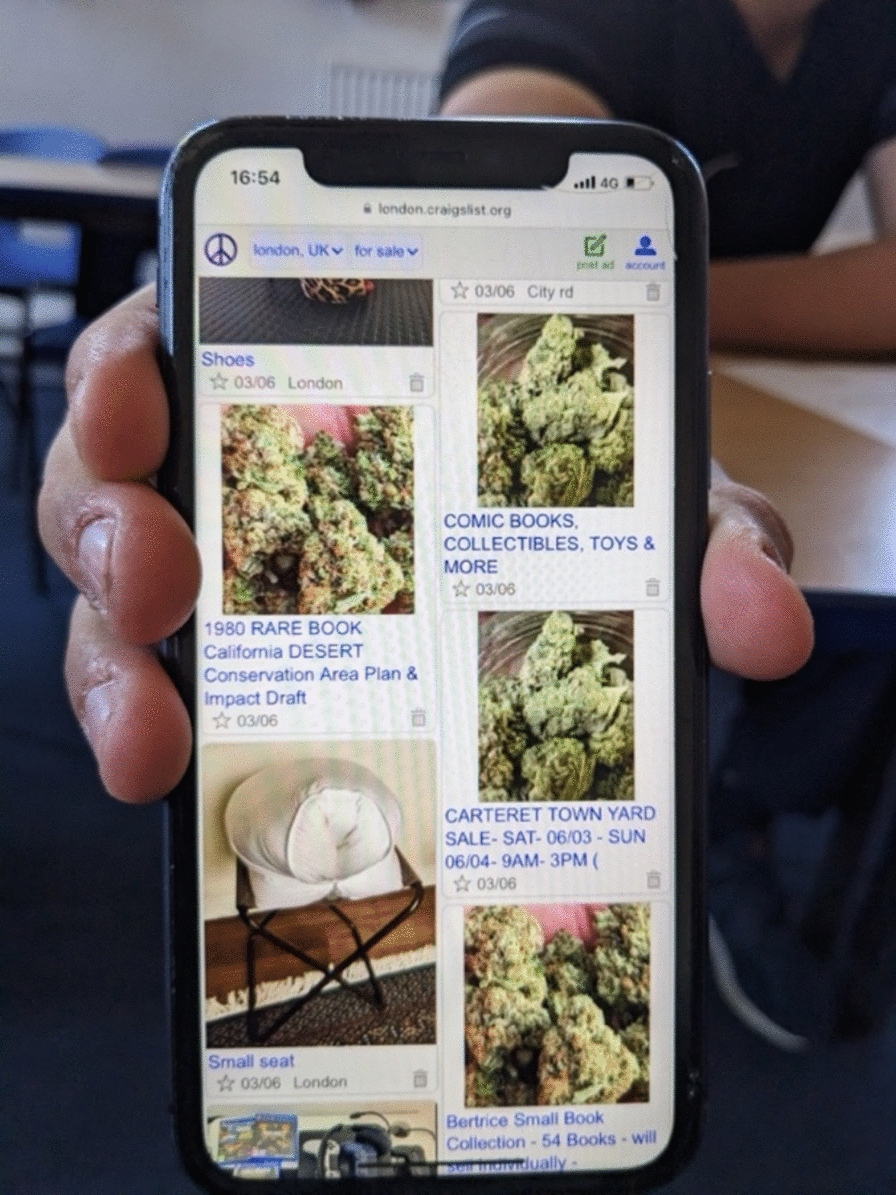
Student participating in the focus group showing the front page of Craigslist.com

Accordingly, the survey was piloted in two stages. First, it was distributed to pupils attending a high school (children aged 13–16) and a further education college (children aged 16–18), from which 88 responses were collected. That survey included a free-text box which encouraged respondents to comment on questions they did not understand or to indicate ways in which the survey could be improved. Secondly, in June 2023, a focus group was conducted by the DSMF and a school teacher with Year 12 students from one school. The session was unstructured to capture insights from the young people’s experiences, with cognitive interviewing used to pretest questions, and to explore participants’ thought processes when they responded [76, 77]. The feedback received from these students was subsequently used to improve the survey questions. Suggested changes included the addition of questions about the platforms *BeReal, Depop, Vinted* and *Craigslist.* For example, as shown in Fig. 1, participants noted that illicit drugs were being advertised on these platforms.

### Participants

Children aged 13–18 were recruited from UK secondary schools between October 2023 and May 2024. A minimum age of 13 was selected to ensure that participants could understand the questions, and to reduce the risk of unethical practices when discussing drugs with younger teenagers. Two substance education charities, DSMF and the Talk About Trust,^1^ helped to distribute the survey through their networks of schools. Students learned about the research during their *Relationships and Sexuality Education* (RSE) or *Personal, Social, Health and Economic* (PSHE) classes. Their teacher explained the aims of the research in class and provided them with information sheets, produced by the study authors. Those interested in participating received a link to an online survey from their teacher and were invited to complete it during class time. They were also given the option to complete it in their own time. Participation was voluntary, and all personal information was anonymised. Participants were required to provide both their own consent and parental consent. The study received ethical approval from the UCL Research Ethics Committee under the *Project ID/Title: 23,897/001: Drugs on Social Media Survey.*

A total of 1443 responses were collected. Responses were excluded if they did not meet the following criteria—providing consent, achieving a minimum completion rate of 70%, and reporting that they used social media. The final dataset comprised 1151 responses. Data analysis was conducted using R.

Table 1 summarizes respondents’ demographics. The average age was 14.7 years (SD = 1.28), with 14- and 15-year-olds being the most common age groups. Year 10 students made up 34% (N = 390) of respondents, followed by 26% in Year 9 (N = 298), while Year 13 was the least represented at 8% (N = 94). Gender distribution was nearly equal, with most participants identifying as either female (51%) or male (47%). Most respondents attended state schools, but there was some representation from other establishments. The survey included 29 schools across 19 counties in England and Scotland, with the largest groups from Moray, Scotland, and Wiltshire, England. Some participants did not provide details of the school they attended.

**Table 1:** Demographic profile of survey respondents

Demographics	Description	Percentage
Age	13	15
	14	34
	15	23
	16	16
	17	8
	18	3
	Total	100
Year group	9	26
	10	34
	11	17
	12	15
	13	8
	Total	100
Gender	Female	49
	Male	46
	Non-binary	2
	Prefer not to say	2
	Total	100
School type	State	75
	Independent	8
	SE/Sixth form college	6
	Faith	2
	Grammar	1
	NA	7
	Total	100
Country	England	40
	Scotland	37
	NA	23
	Total	100

## Results

The results are structured as follows: the first section provides descriptive findings about students’ experiences with drugs on social media including their use, exposure to drug-related content, and perceptions of drug availability, quality, and associated risks. It also covers practical aspects, such as the apps used for purchasing drugs. The second section examines the relationships between participants exposure to drug advertisements and their attitudes and behaviours and tests for H1 and H2.

### A snapshot of school students’ experiences about drugs on social media

#### Characterising drug portrayals on social media: content types and substances involved

##### Types of content related to drugs

Participants were first asked if they had seen drug-related content, excluding illicit drug advertisements, across the social media platforms they use. For clarity, ‘*drug-related content seen overall*’ refers to all drug-related material encountered on social media and messaging platforms, including memes, educational posts, depictions of use, legal promotions, challenges, and news stories. ‘Illicit drug advertisements’ refers specifically to content promoting the sale or purchase of illegal substances. Sixty percent (N = 680) reported encountering drug-related content, including news, educational posts, challenges, and others about consuming drugs. As shown in Table 2, the most frequently reported types of drug-related content were memes and content intended to be amusing. Educational content followed, while content depicting other people consuming drugs was also frequently seen.

**Table 2:** Number and percentage of participants who reported seeing each type of drug-related content on social media (N = 680)

Content type	Number of participants seeing content $\ge 1$ across their social media platforms	%	Total mentions across platforms
Memes/Funny	459	40	1495
Educational	358	31	885
Others consuming	315	27	731
Legal ads	208	18	503
Challenges or Tricks	173	15	402
News	143	12	400

Participants could select more than one content type. Counts reflect the number of unique participants who reported seeing at least one instance of each type of drug-related content across any social media platform. Percentages are based on the 680 participants who reported having seen drug-related content

**Fig. 2:**
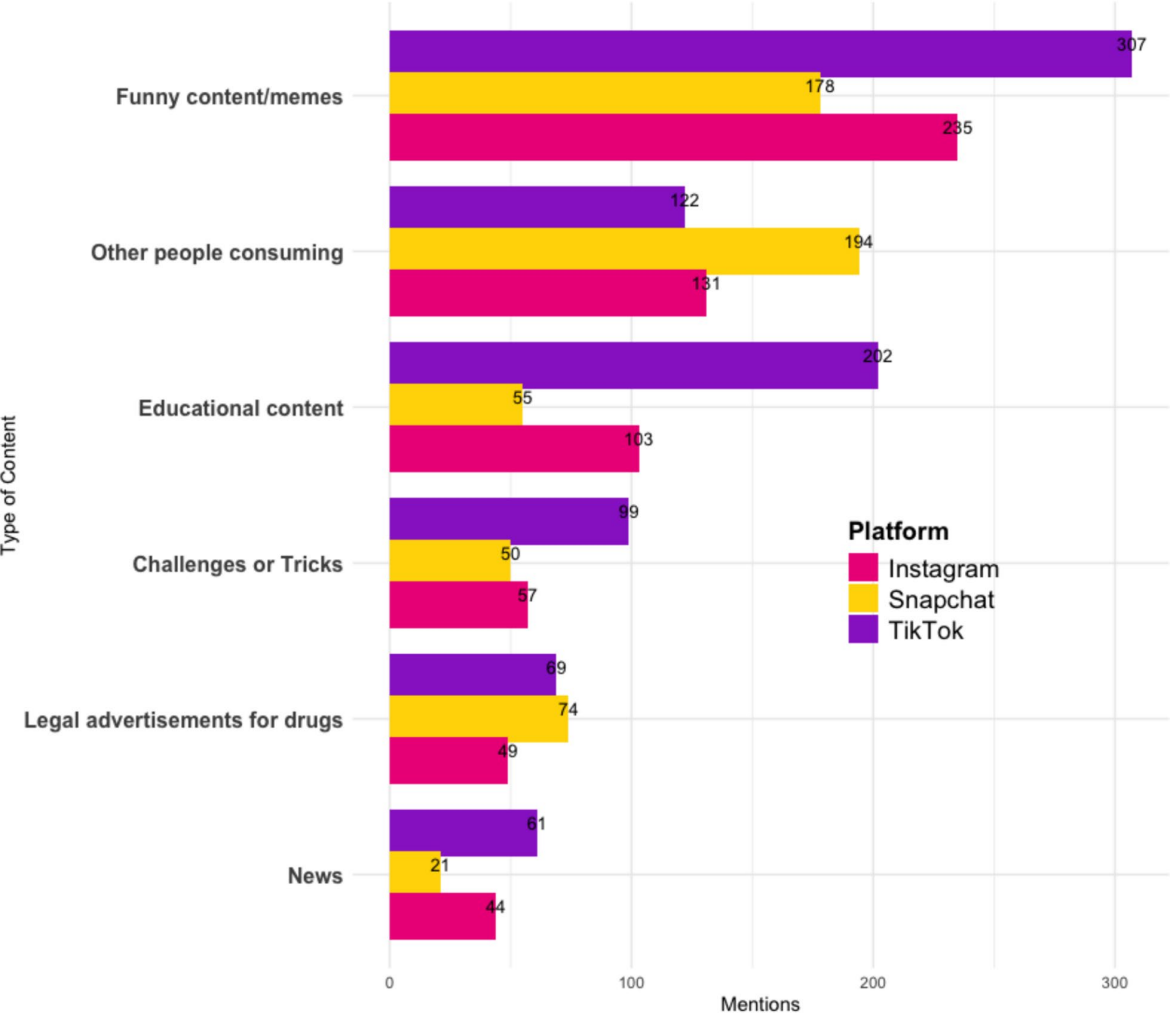
Types of drug-related content seen among the 3 main social media platforms (Instagram, Snapchat and TikTok)

Across all types of drug-related content, TikTok, Snapchat, and Instagram were the most frequently mentioned platforms overall, although their prominence varied slightly depending on the specific content type (see Fig. 2). These platforms, widely popular among younger demographics, appear to serve as key channels for the distribution of drug-related content. However, the nature of the content seen on these platforms varies. For instance, educational content was more frequently reported for TikTok than for Instagram or Snapchat. While humorous content and memes were more frequently observed on both TikTok and Instagram, on Snapchat, relative to the other platforms, participants reported viewing content depicting others consuming drugs more frequently. Legal drug advertisements were reported at comparable rates on TikTok and Snapchat, both of which were higher than on Instagram.

##### Seeing illicit drug advertisements

More than half of young people surveyed (64%, N = 721) reported never seeing illicit drug advertisements on their social media feeds. However, 29% of them (N = 324) indicated that they had seen such advertisements while 7% participants (N = 74) chose not to answer this question.^2^ Respondents who reported that they had seen drug advertisements on social media had a mean age of 15.15 (SD = 1.28), which was significantly higher than the mean age for those who had not (14.58, SD = 1.24), ($t(604.5) = - 6.72,\;p < 0.001$) using Welch’s *t*-test due to unequal variances. Among those who reported that they had encountered illicit drug advertisements, only 5% (N = 16/324) stated that they had actively searched for them. In contrast, 83% (N = 268/324) reported that the advertisements just appeared on their social media feed, while 12% (N = 38/324) reported that they had encountered them both passively and by searching for them.^3^ This suggests that, for most young people, illicit drug advertisements on social media appear organically on their social media feed.

##### Types of drugs seen overall vs. in illicit ads

**Fig. 3:**
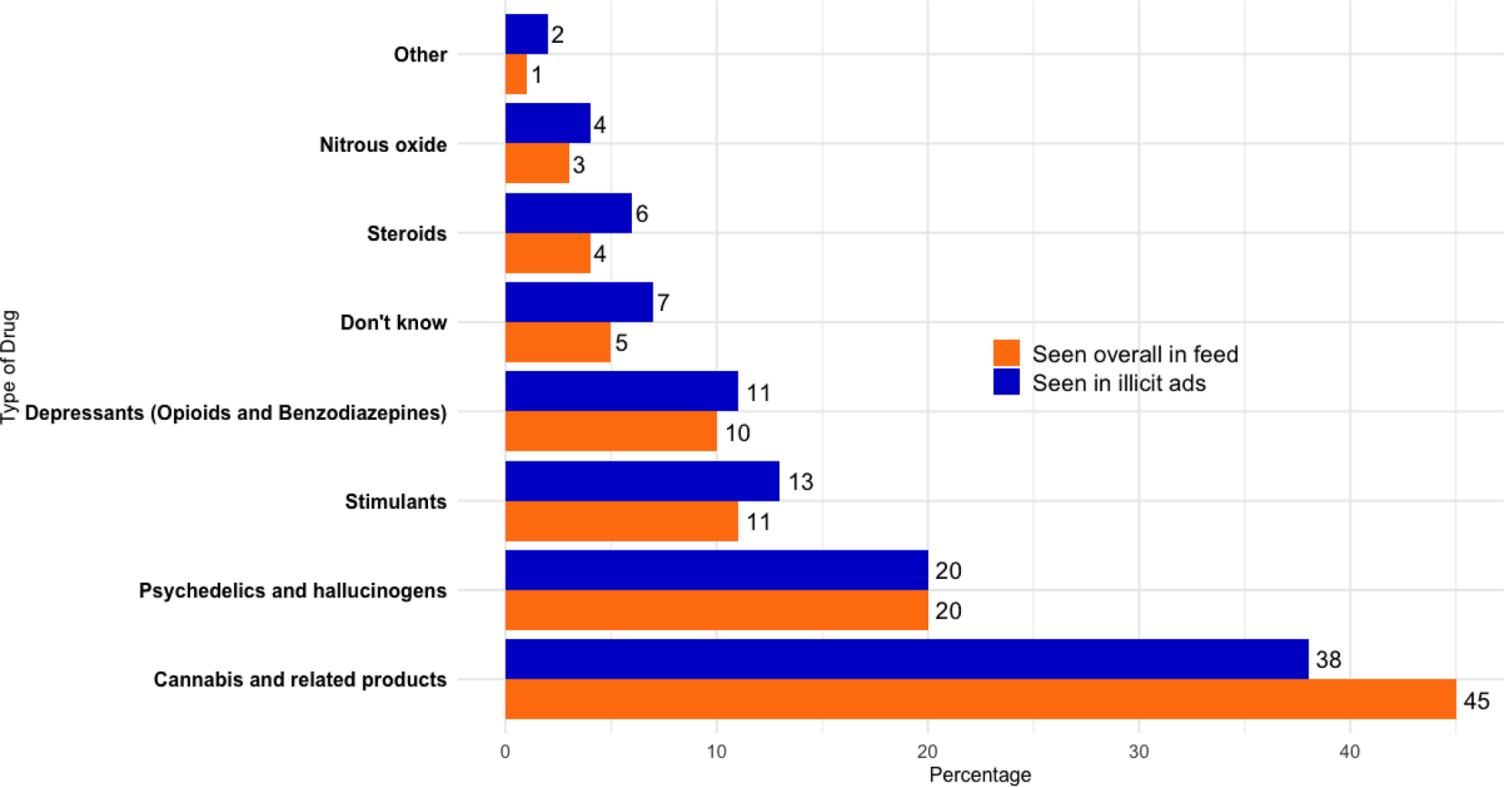
Comparison of types of drugs seen overall in participant’s social media feed vs. specifically in illicit drug advertisements

As shown in Fig. 3, cannabis and cannabis-related products were by far the most frequently observed type of drug content reported by young people on social media, accounting for 45% of drug-related content seen in their feed and 38% of illicit drug advertisements. These included products such as cannabis oil, THC vape liquids, edibles and synthetic cannabinoids. Psychedelics and hallucinogens (incl. Psilocybin mushroom, LSD, MDMA and Ketamine) were the next most mentioned type of drug in both young people’s overall feed and for illicit drug ads. Stimulants (Cocaine, Amphetamines, Mephredone), Depressants^4^ (including opioids such as heroin, codeine, and tramadol, and benzodiazepines such as Valium and Xanax), Nitrous Oxide and Steroids were more frequently seen in illicit drug ads rather than overall in social media’s feed. In the category ‘Other’, young people reported seeing drugs including Fentanyl (4 mentions), ‘Tranq’ (Xylazine) (2), Adderall (1), Cough sweets (1), Calpol (Paracetamol for infants) (3), New Psychoactive Substances (NPS) (2), ‘Monkey dust’ (MDPV) (2), and there was one mention of ‘Foxy methoxy’ (5-MeO-DIPT). The ‘Don’t know’ category, with 191 mentions, suggests some uncertainty or lack of specific knowledge among respondents.

Except for cannabis and psychedelics, most types of drugs were somewhat more likely to be seen in illicit drug advertisements than in other types of content. This perhaps reflects growing discussions in the UK regarding cannabis policy and decriminalisation [78, 79], as well as the decriminalisation of cannabis in other countries. Although policy discussions about psychedelics are not at the same stage, emerging research about the therapeutic benefits of them [80–82] may have contribute to the increased frequency with which they are included in forms of social media content that are not limited to drug advertisements.

#### Seeing drugs on social media: the role of platforms

**Fig. 4:**
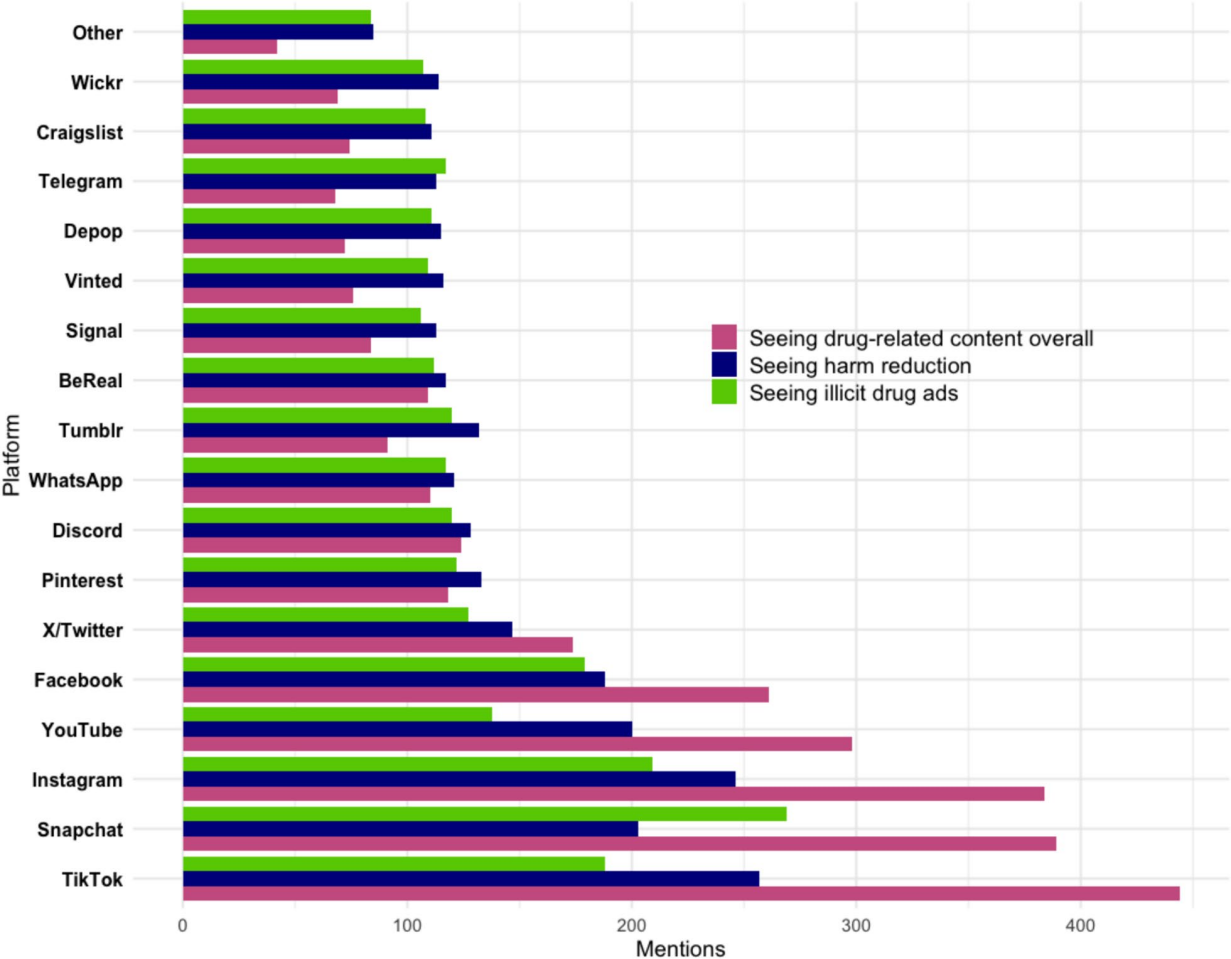
Seeing drug-related posts, illicit drug ads and harm reduction/drug safety advice posts by platform

Participants were asked about the social media platforms on which they have encountered drug-related content, including illicit drug advertisements and harm reduction advice. Figure 4 shows that the most frequently mentioned platforms for seeing illicit drug advertisements were Snapchat, Instagram, and TikTok. Specifically, of the 324 students who reported seeing illicit drug ads on social media, 83% encountered them on Snapchat, 65% on Instagram, and 58% on TikTok. Following these, Facebook was reported by 55% of participants, YouTube by 42%, and X (formerly Twitter) by 39%. The least mentioned platforms included Craigslist (33%), Wickr (33%), and Signal (32%). Interestingly, platforms not typically thought of as social media, such as Depop, Vinted, and Craigslist, were also reported as having included such content, suggesting initial evidence of their use for drug advertising and sales. Additionally, platforms like Pinterest (38%) and Tumblr (37%), which are less popular or have declined in usage among young people, were still cited as significant venues for drug advertisements.

Of the total sample (N = 1151), 77% answered the question regarding exposure to harm reduction advice. Within this responding group, 51% reported seeing substance safety advice in their feeds and 43% reported not seeing such advice. The remaining respondents either did not answer or preferred not to say. TikTok and Instagram emerged as the most frequently mentioned platforms for encountering harm reduction advice, with 67% of students who saw this type of advice reporting it was on TikTok and 64% indicating they saw it on Instagram.

Some of the less frequently mentioned platforms among young people, such as Wickr, Craigslist, Telegram, Depop, Vinted, Signal, and Tumblr, were nevertheless mentioned equally or slightly more often for seeing harm reduction advice compared to illicit drug ads —maintaining a balance between the two, while overall drug-related content was comparatively less referenced. This may be explained by the fact that online drug sellers often provide tailored harm reduction and safety advice alongside their drug sales [1, 83]. In contrast, Snapchat stands out as an outlier, where illicit drug ads and overall drug-related content significantly surpass harm reduction advice compared to TikTok.

##### Comparing platform exposure of illicit drug ads: Does viewing frequency matter?

**Fig. 5:**
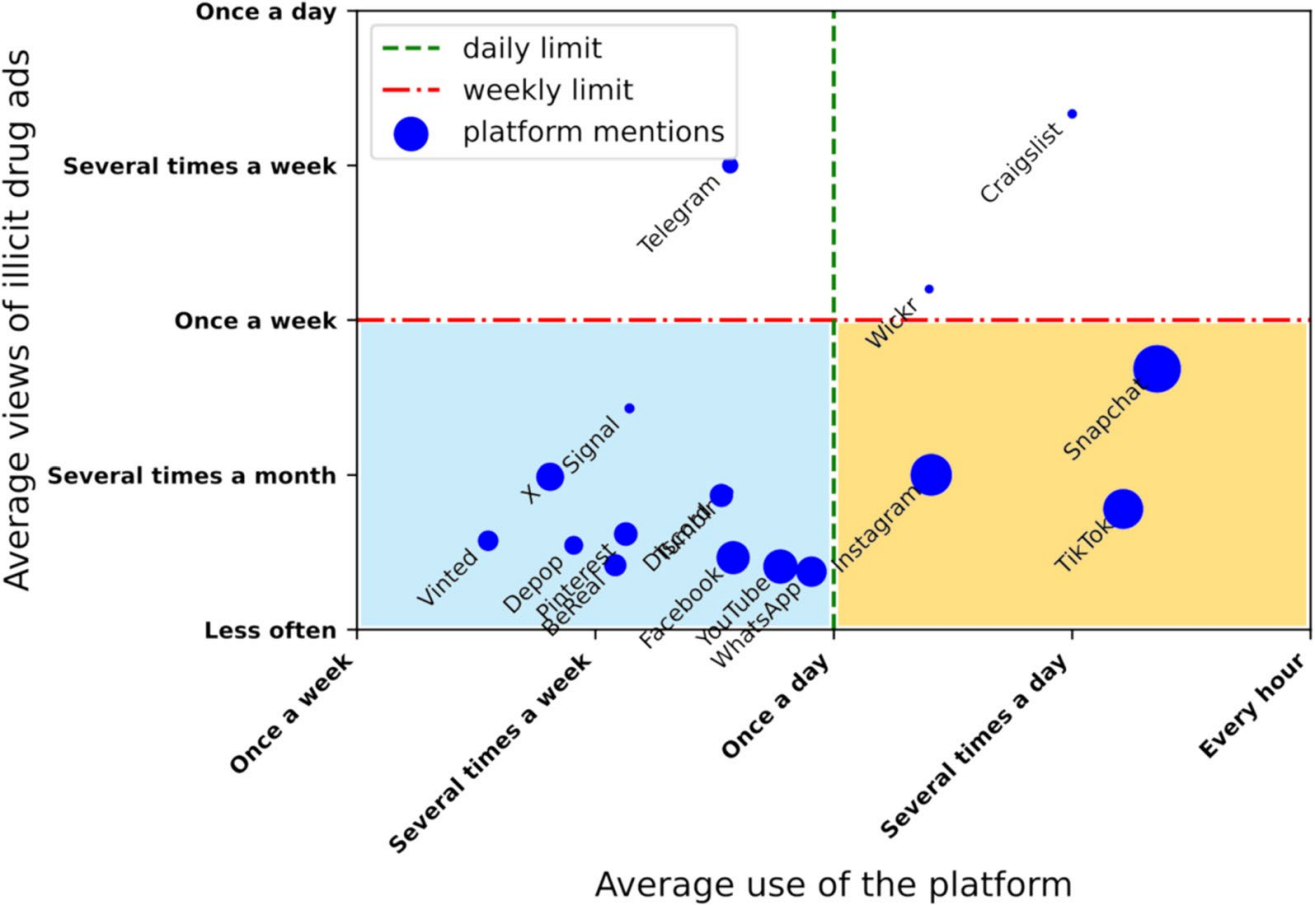
Average visibility of illicit drug ads by platform compared to participants’ average use. The lower left quadrant (in blue) represents platforms with low-use and low exposure, the lower right quadrant (in orange) represents the high-use, moderate exposure platforms and the upper quadrant (in white) represents the platforms with low-use and high exposure. The superimposed data points in the blue quadrant are platforms ‘Discord’ and ‘Tumblr’. In addition to the average, the median views and use were calculated to account for the assumption of proportionality between categories (See Fig. 7)

Participants were asked about the platforms on which they encountered illicit drug advertisements and the frequency of both the rate at which they encountered them and their use of those platforms. Figure 5 shows the rate with which participants reported exposure to illicit drug advertisements for each platform (y axis) against their use of the same platform (x axis).

Platforms can be segmented into three main categories:

- (1) *Low-Use, Low Exposure*: Platforms used weekly where drug ads are seen infrequently (monthly) (lower left quadrant in blue)
- (2) *High-Use, Moderate Exposure*: Platforms used daily with drug ads seen on a monthly to weekly basis (lower right quadrant in orange)
- (3) *Varying-Use, High Exposure*: Platforms used by few participants but where drug ads are seen more than once a week (upper quadrants in white)

In Fig. 5, the largest group of platforms, including WhatsApp and Vinted, were used less than once a day and illicit drug advertisements were observed much less frequently than this. This group might be considered relatively ‘low use and low exposure’ to illicit drug advertisements. A second group of platforms comprised the “big three” (Snapchat, Instagram, and TikTok) and was characterised by frequent use (more than once a day), but the rate at which illicit drug advertisements were encountered was also below the weekly threshold. However, there are notable differences in exposure to drug advertisements for the two most frequently used platforms, despite participants reporting that they used them with a comparable frequency.

Finally, for three platforms (Craigslist, Telegram, and Wickr), participants reported encountering illicit drug advertisements more than once a week. Craigslist and Telegram had the highest average reported frequency of drug advertisements (several times a week), though Craigslist is used daily, whereas Telegram is not. For all three platforms, the number of participants that reported using them was small (N = 6 for Craigslist and N = 23 for Telegram). However, those that reported using them were from different schools across England and Scotland, suggesting that seeing drugs on these platforms was not an isolated or geographical phenomenon. It is possible that while users on platforms like TikTok or Instagram are more likely to unintentionally encounter drug advertisements due to their broad user bases, those reporting drug sales on Craigslist or Telegram may be engaging more intentionally, as these platforms are known for facilitating such transactions. Interestingly, there was no mention of the UK selling platform Gumtree being used to advertise illicit drugs, whereas Craigslist, a similar platform commonly used in the US, was mentioned. Depop and Vinted, which also enable selling, but for which rates of exposure were low, may use computer vision and automated systems to monitor listings (e.g. they do so to classify items sold), which would result in lower exposure to illicit content. In contrast, platforms like Craigslist, that is not known to use machine vision in the same way, may have higher exposure to such ads. Future research might investigate this explicitly.

#### Buying illicit drugs on social media

When asked about buying drugs through social media, 82% of respondents (N = 871) said they had not done so, while 10% (N = 109) reported that they had (about 8% (N = 81) chose not to answer). Table 3 summarises the delivery and payment methods used by participants to purchase illicit drugs on social media. When answering these questions, participants could select more than one answer and hence the percentages do not sum to 100%.

**Table 3:** Reported delivery and payment methods used when buying drugs through social media (N = 109)

% Respondents					
Apps used to organise delivery		Delivery method		Payment method	
Snapchat	64	Arranged place	49	Cash on delivery	68
Text message^a^	33	Don’t know	31	PayPal	22
Instagram	29	By a friend	28	Bank transfer	22
Telegram	17	Delivery at home *(in person)*	18	Don’t know	21
Don’t know	14	Through the post *(mail)*	13	Cash app	15
WhatsApp	13	Other	6	Cryptocurrency	12
TikTok	13			Online gift card	6
Other	6			Other	5
Facebook	6			MoneyGram	5
X	6				
Tumblr	5				
YouTube	3				
Wickr	1				

^a^Text message (SMS) was included as a response option due to its role in drug delivery communications and continuity with the source survey. While not classified as a social media platform, it was retained in this analysis to reflect actual youth practices. Excluding participants who only reported SMS (N = 36) does not meaningfully alter the overall proportions

Young people reported using different social media platforms to coordinate drug deliveries. However, Snapchat was the platform most commonly used, accounting for more deliveries than the other platforms combined. Tumblr, YouTube, and Wickr were rarely reported to be used, despite other research suggesting that Wickr is a popular platform for young adults organising illicit drug deliveries [1, 6]. This discrepancy may reflect both evolving social media trends across age groups and the discontinuation of Wickr in December 2023 [84].

In terms of the delivery of illicit drugs, young people reported using a variety of methods, but the most common was for the dealer to arrange delivery to a specific location. A non-trivial amount reported having drugs delivered to their home, either by a dealer or through the post. With respect to payment, most young people used cash on delivery, although PayPal and bank transfers were also relatively popular. While initially surprising given the young age group, 12% of young people reported using cryptocurrencies to pay for drugs. However, this figure aligns with broader trends as a 2023 survey of 13- to 16-year-olds in the UK indicated that 8% had already invested in cryptocurrencies [85].

### Understanding the relationship between exposure to drug advertisements on social media and the attitudes and behaviours of young people

This section examines the associations between exposure to illicit drug advertisements on social media and young people’s attitudes and behaviours.

#### Risk perceptions and feelings around seeing drugs on social media

**Fig. 6:**
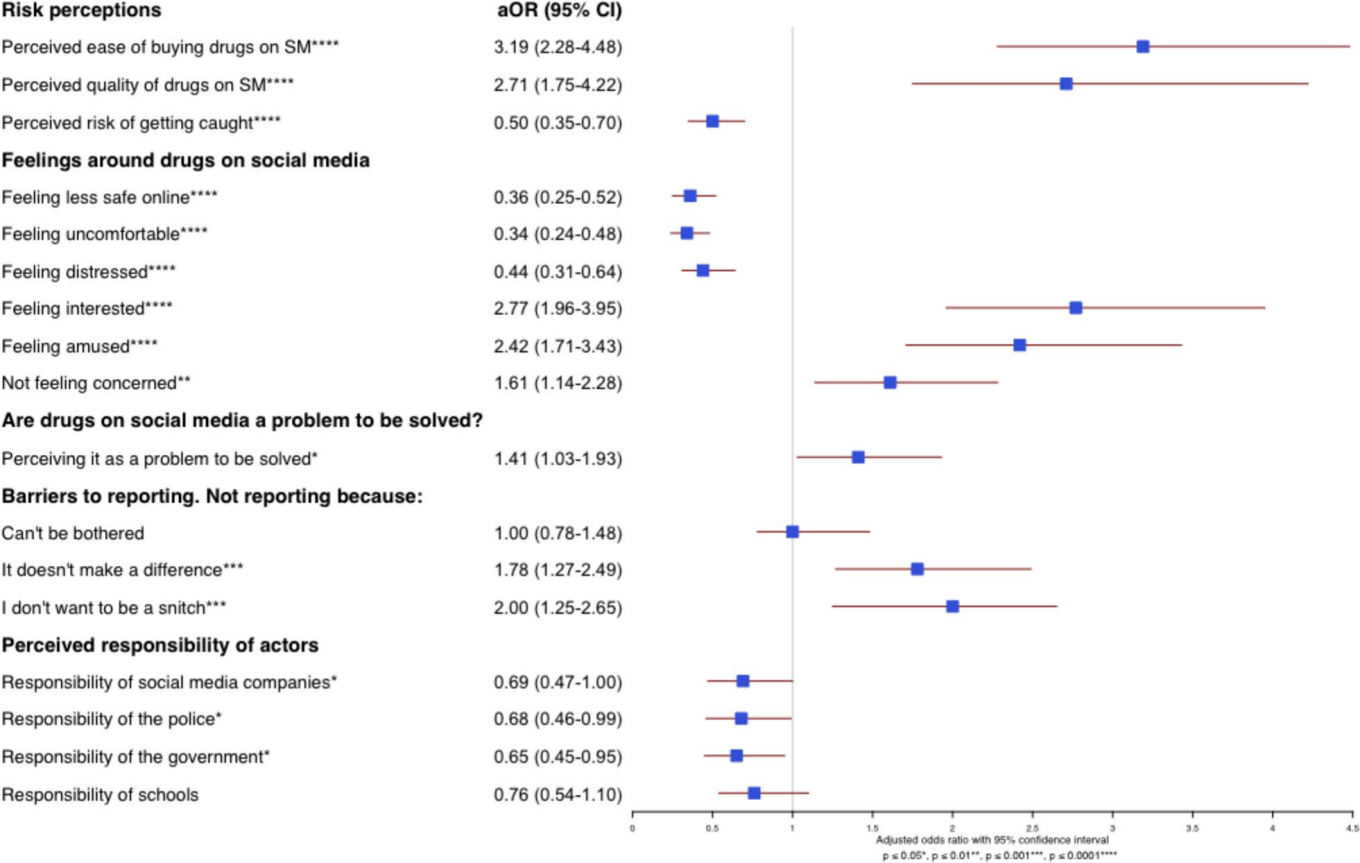
Relationship between exposure to illicit drug advertisements and dependent variables: adjusted odds ratios for risk perceptions, feelings, barriers to reporting and perceived responsibility of actors. The dependent variable ‘buying drugs on social media’ was excluded from the forest plot as its high odds ratio (17.39) falls far outside the chart’s range, making it unsuitable for visualisation. Details of this model are provided in Appendix Table 6

**Fig. 7:**
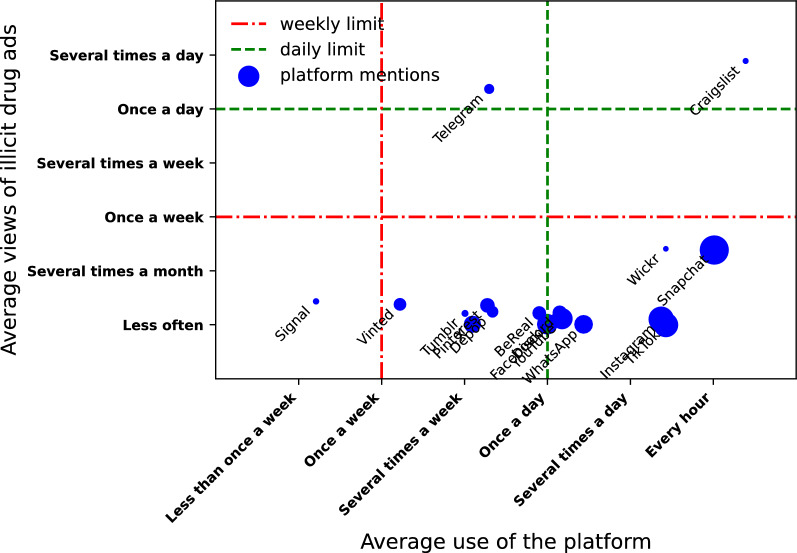
Median visibility of illicit drug ads by platform compared to participants’ average use

As shown in Fig. 6, individuals who had seen drug advertisements on social media had significantly higher odds of perceiving it as easier to buy drugs, of believing that drugs advertised on social media are of higher quality than those sold on the street, and lower odds of thinking they would get caught buying them, providing support for H1 (see Table 4).

When it comes to feelings about seeing drugs on social media, exposure to illicit drug advertisements is associated with significantly lower odds of young people reporting that they feel unsafe, uncomfortable, or distressed (Fig. 6). However, exposure to illicit drug ads is significantly associated to young people reporting they are or would be interested in the advertisement, being amused by it and not feeling concerned about this issue (See Table 5b).

#### Seeing is buying: the impact of illicit drug ads on purchasing behaviour

The relationship between exposure to illicit drug advertisements and the purchase of illicit drugs on social media has not been previously explored in the literature. The results in our sample provide support for H2, showing a positive association between seeing illicit drug ads on social media and purchasing illicit drugs through these platforms $(OR=17.39, B = 2.86, SE = 0.34, p < 0.0001)$ while controlling for age, gender, school year, country and social media use (Table 6). Participants exposed to illicit drug ads were more likely to have bought drugs on social media compared to those who had not seen such content $(95\% CI: 9.27, 35.42)$. The R^2^ value of 0.287 (Table 6) indicates a good fit of the model [86] in explaining the variation in drug purchasing behaviour. While the relationship may appear intuitive, these findings help to quantify the strength of association and underscore the relevance of content exposure in shaping online drug-related behaviours.

#### Reporting practices and perceptions: Is it really a problem?

When asked if the presence of drugs on social media was as a problem that needs to be addressed, about half (49%, N = 460) believed that it was—only 24% (N = 226) did not view it as a problem (27%, or N = 254 were uncertain). However, those who had encountered illicit drug ads had 1.41 times the odds of viewing such content as problematic compared to those who had not (Fig. 6).

When asked if they had reported (or would do so if they encountered) illicit drug advertisements on social media, 87% (N = 908) indicated that they had (or would) not. This aligns with previous research showing that only a small proportion of young people report harmful online content [27, 87, 88]. Only 7% (N = 73) reported that they had or would report illicit drug adverts encountered, while 6% (N = 66) chose not to answer. Young people were also asked about the primary reasons they had not or would not report illicit drug advertisements. The three main barriers identified were:

- Not being bothered to report. This was the primary reason for those who had seen drug adverts (24%, N = 145) and those who had not (23%, N = 311),
- Believing that reporting would be ineffective (20%, N = 121 and 16%, N = 214, for those that had and had not encountered illicit drug advertisements, respectively),
- And avoiding the stigma of being labelled *‘snitches’* (16%, N = 93 and 11%, N = 147, for those who had and had not encountered illicit drug advertisements, respectively).

Further analyses were conducted to see if exposure to illicit drug advertisements was associated with differences in perceptions of, or thoughts about, the main barriers to reporting. Analyses showed that having encountered an advertisement (or not) was unrelated to whether they could be bothered to report an advert. However, as highlighted in Fig. 6, young people exposed to drug ads on social media had 1.78 times greater odds of refraining from reporting because they believed it wouldn’t make a difference, and twice the odds of avoiding reporting for fear of being seen as a ‘snitch,’ compared to those who hadn’t seen any advertisements (see also Table 8).

#### Who do young people believe should be responsible?

Students were asked to what degree (ranging from ‘responsible’ to ‘not responsible’) they believed that social media companies, the police, government, and schools should be responsible for stopping illicit drug advertisements on social media platforms. About 80% of respondents felt that social media companies, the police and the government were either responsible or somewhat responsible. In the case of schools, this figure was slightly lower (70%), but most respondents clearly felt that all of these organisations were responsible to some degree. However, this perception was affected by whether respondents had seen illicit drug advertisements or not. Figure 6 show that having seen illicit drug ads on social media was significantly associated with perceiving social media companies as having less responsibility to solve this problem as well as the police and government (See also Table 9).

#### The influence of control variables

The control variables included in the models were used to increase the robustness of the study and consequently, for the sake of brevity, we have note focused on them in the text above. However, some interesting trends emerged, which we discuss here. Overall, individuals who use social media more frequently tend to have lower risk perceptions and fewer negative feelings regarding drugs on social media. Relative to users who engage with social media less than once a day, more frequent social media use was also associated with the perception that drugs are easier to obtain (*OR* = 0.21, *B* = 1.54, *SE* = − 0.51, *p* < 0.001) (Table 4). The most frequent social media users also had lower odds of reporting feeling uncomfortable or concerned about drugs on social media. Hourly social media use (compared to using social media less than once a day) was associated with 0.39 times the odds of having purchased illicit drugs via social media (*OR* = 0.39, *B* = − 0.95, *SE* = 0.29, *p* < 0.0001) (Table 6). However, these users had lower odds of perceiving this as a problem, though they had greater odds of believing that actors such as social media companies, the police, the government, and schools/parents were responsible for addressing the issue of drugs on these platforms (Tables 7, 8, 9).

An association with gender also emerged as significant in many cases. Males had lower odds of reporting feeling distressed when encountering drugs for sale on social media and were half as likely (0.5 times the odds) to have purchased illicit drugs through these platforms (Tables 5a, 6), perhaps signalling that females prefer to buy online due to safety concerns. Furthermore, males had 0.69 times the odds of reporting illicit drug advertisements due to concerns about being perceived as a ‘snitch’ (*B* = − 0.38, *SE* = 0.18, *p* < 0.05), suggesting that gender stereotypes may influence reporting behaviours (Table 8).

Age and school year were, in some cases, significantly associated with the dependent variables. For example, students in Year 11 had 0.52 times the odds of believing they would get caught buying or selling drugs on social media (*B* = − 0.66, *SE* = 0.27, *t* = − 2.42, *p* < 0.01). They also had 2.81 times greater odds of seeing the police (*B* = 1.03, *SE* = 0.31, *p* < 0.001) and 2.02 times greater odds of seeing the government (*B* = 0.70, *SE* = 0.30, *p* < 0.01) as responsible for addressing the issue (Tables 4, 9). In contrast, students in Year 9 had 1.78 times greater odds of feeling unsafe online (*OR* = 1.78, *B* = 0.58, *SE* = 0.25, *p* < 0.01) and 1.96 times greater odds of feeling uncomfortable in response to illicit drug advertisements on social media more uncomfortable (*B* = 0.68, *SE* = 0.25, *p* < 0.01) in response to illicit drug advertisements on social media (Table 4).

## Discussion

The results from our survey highlight one undeniable fact: drugs are a visible part of young people’s social media environments and online routines. With 60% of participants reporting that they saw drugs on their feed, encountering substance-related content on social media is neither rare nor occasional. Most of this content is humorous, followed by educational content and posts showing others consuming drugs.

The prevalence of illicit drug advertisements also appears to be substantial, with 29% (324 students) stating they had encountered such ads on their social media feeds. Of these, 83% saw the ads without actively searching for them. Despite not being a representative sample, this figure echoes Ofcom’s finding that 32% of young people reported seeing harmful content online in the past year, with exposure increasing with age [89].

While the platforms commonly thought to host illicit drug advertisements (Snapchat, Instagram, Facebook) were identified in our survey, we also provide initial evidence of that emerging platforms, not traditionally considered to be social media, are becoming relevant for the sale of illicit drugs. These include clothing resale apps such as Depop and Vinted, but also the use of Discord servers, previously identified for drug sale in New Zealand [15]. Young people’s responses also highlight platforms that may have been overlooked and are often regarded as less current, such as Tumblr and Pinterest.

Our study provides a novel contribution to the literature by showing that exposure to illicit drug ads on social media significantly shapes young people’s risk perceptions and attitudes. We found that those exposed to such ads were about three-times more likely to view drugs as easier to buy, and to believe that such drugs were of higher quality than those available on the street, and about 50% less likely to perceive a risk of getting caught. These findings are consistent with Hypothesis 1. In line with Hypothesis 2, we also find that young people exposed to illicit drug ads were 17.39 times more likely to report that they would purchase them, suggesting a strong association between ad exposure and purchasing intentions.

Where does this leave us? The breadth of data calls for a critical reflection on the implications of these findings to effectively address the sale and advertisement of illicit drugs on social media. Here, we focus on three key aspects: first, the significance of platform exposure and its implications for regulatory measures; second, whether the impact of exposure to drug advertisements is associated with a process of desensitization; and finally, potential opportunities to leverage social media as a tool for implementing protective systems and barriers against harmful drug-related content.

### Providing a more granular understanding to platform exposure to illicit drug ads

Previous studies have shed light on the different types of platforms commonly used to advertise and sell illicit drugs: notably Snapchat, Facebook, Instagram [1, 5, 6, 39, 44], TikTok [40, 42, 90], X (formerly Twitter) [91–93], Wickr, Telegram and even Discord [15, 49, 94, 95].

This body of literature provides valuable insights into how digital platforms are used by buyers and sellers, methods for advertising, and the interplay between these practices and user preferences, motivations, and offline markets. However, the relationship between the frequency of exposure to drug ads and platform usage was previously underexplored. Although a positive correlation between social media usage and the likelihood of exposure to drug-related content seems intuitive—an association initially identified McCulloch & Furlong ([6], p.19)—emerging evidence raises questions about the linearity of this relationship. Research suggests that individuals involved in the sale or purchase of drugs on social media engage in these activities for diverse reasons, often selecting platforms based on specific features [1, 5], cultural identities and affiliations [21, 23] or their integration within offline networks [96].

This prompts the question of whether some platforms are more frequently used as primary channels for purchasing drugs due to their inherent features, or simply because of their widespread popularity. Additionally, are there platforms that, while only occasionally used or considered more ‘niche’, consistently display illicit drug advertisements? Estimating the prevalence of illicit drug advertisements on social media platforms presents significant challenges due to the absence of standardized baseline measures or comparable data across different platforms and drug types. This challenge is exacerbated by the fact that most social media platforms do not authorise data collection for independent research purposes, and when they do, access can be prohibitively expensive. Financial cost remains a primary barrier to researchers seeking to engage in platform transparency work [97]. This lack of accessibility further hinders efforts to quantify the volume and spread of illicit drug advertising across platforms and the ability to make reliable comparisons [8].

Among the three most popular platforms in our sample (Snapchat, TikTok, and Instagram) there were notable differences in the visibility of drug ads based on usage frequency. Snapchat shows drug ads more often than TikTok and Instagram, with ads appearing about once a week compared to just a few times a month on the other two platforms. Conversely, despite there being similar reported rates of exposure to illicit drug advertisements for Instagram and TikTok, people use TikTok more often. This raises the question of whether the frequency with which drugs are advertised varies across these platforms, or if TikTok’s proactive detection system is more effective at identifying illicit drugs than those employed by Snapchat and Instagram. These findings offer an initial, granular understanding of exposure and usage patterns related to illicit drug advertisements across various platforms, while also shedding light on the effectiveness of content moderation processes. This represents a significant first step in evaluating proactive detection methods, though further research is needed to draw more definitive conclusions.

Proactive detection systems are central to social media companies’ strategies for addressing illegal content online and meeting the demands of the UK’s online safety regulatory framework [28]. From April to June 2024, Meta reported that it proactively detected 97.90% of violating content related to restricted goods and services on Faceboo] and 99.60% on Instagram, with only 0.40% of Instagram users reporting such violations [98]. In its transparency report, Snapchat noted that globally, it enforced actions against 241,227 pieces of violating content related to drugs across 166,562 unique accounts resulting in a Violative View Rate (VVR) of 0.01% [99]. This means that out of every 10,000 views of Snaps and Stories, only one contained content violating their policies. However, given the volume of content posted, it remains an open question as to how satisfactory these figures are, and what potential discrepancies exist between how platforms define and report violating content and young people’s perceptions of illegal drug content? Comparable figures for TikTok are not publicly available, as its transparency reports do not provide specific metrics on drug-related content or use different categorisations that limit direct comparison. These inconsistencies raise important regulatory implications, particularly concerning how platforms with limited capacities or smaller user bases can implement effective moderation systems and whether their effectiveness can be evaluated in the same manner.

### The impact of seeing illicit drug advertisements: a process of normalisation and desensitisation, or neither?

Based on our survey, seeing posts advertising illicit drugs on social media was associated with perceptions that these substances as safe, easy to obtain, funny, and either interesting or not particularly concerning. But do such differences in risk perceptions stem solely from the increasing social acceptance of this content, or could other individual processes be at play when individuals are repeatedly exposed over time to such ads? Desensitisation, in its more clinical definition, refers to the process of repeated exposure to emotional or physiological stimuli to reduce an individual’s response to it. This concept has been applied as a therapeutic technique to reduce anxiety and phobias for decades [100], but it has also been used more broadly in public health and media studies to explain the reduced emotional impact on individuals of repeated exposure to violent or harmful content [101–103]. The process of desensitisation to harmful content has been widely studied and debated in relation to violence and video games and shows mixed evidence [104–108]. However, the literature on individuals working in content moderation for social media platforms may be more relevant in demonstrating that repeated exposure to distressing content can also lead to desensitisation [109–112]. Future research should consider whether exposure to other types of harmful content, such as violent, sexual or self-harm material, may contribute to young people feeling less concerned when encountering drug-related content, perceiving it as ‘less harmful’ in comparison.

Can illicit drug ads on social media be said to contribute to the normalisation and desensitisation of drugs among young people? Perhaps, but such claims require caution. Similar to the debate on violence desensitisation in gaming, which is riddled with methodological flaws [113, 114], these assertions must be carefully scrutinised. It is crucial to distinguish between exposure to harmful content and its potential to cause direct harm. Our survey reveals a disconnect between young people’s indifference towards drug-related content and their recognition of its problematic nature, pointing to a knowledge gap. While our findings are correlational rather than causal, they align with expectations that exposure does not necessarily translate into concern, highlighting the need for further research to clarify this relationship. Perhaps the normalisation debate of the mid-1990s is reflected here, suggesting that while drugs are more widely present online, this does not equate to a generalised endorsement but instead contributes to the cultural knowledge of being ‘drugwise’ [33, 34]. Avoiding moralistic narratives about illicit drugs on social media will be essential for moving beyond polarised debates about its harms. This approach can foster further research to identify effective strategies for reducing harmful illicit drug content online and leveraging social media as a tool to prevent and address such content. The challenge will lie in balancing the protection of young people from harmful content with facilitating open discussions about drugs and drug safety.

### Working with, not against social media: the need for an online safety approach

In addition to highlighting concerns about exposure to illicit drug ads on social media, our findings showing that drug safety advice is frequently seen on some platforms by young people also reveal the positive role social media can play in providing educational and harm reduction content. This underscores the need to rethink how research on drugs and social media is framed.

The shift from social media as a ‘platform space’ to an ‘immersive experience’ highlights the need for more flexible and nuanced approaches when studying these environments [115–118]. Drawing on [119] use of [120] metaphors of *tool, place,* and *way of being;* social media can be understood not merely as a medium for interaction but as an integral part of everyday life. This framing is especially relevant considering our findings, which show that while young people are exposed to illicit drug advertising on social media, they also frequently encounter drug safety advice and harm reduction content. In this context, social media is not only a potential source of harm but also a tool for support, learning, and even protection [61, 121, 122]. Understanding social media as a ‘way of being’ may help moving beyond binary notions of good or bad platforms. This perspective invites a reconsideration of how research on drugs and social media is framed, suggesting a shift towards approaches that better account for young people’s embedded, everyday experiences. It also highlights their expectation that both platforms and police should take responsibility for addressing drug-related content online, with clear implications for regulatory oversight and the implementation of the Online Safety Act.

Looking ahead, the regulatory and legal landscapes surrounding both drugs and digital platforms may evolve in ways that significantly affect young people’s online experiences. For instance, policy changes that move currently illicit substances like cannabis or psychedelics into more regulated or legal markets could introduce new challenges around age-restricted advertising and the visibility of drug-related content for users under the legal age of purchase. Similarly, proposals to restrict or ban social media use for under-16 s, as recently legislated in Australia and proposed in the UK [123] may reshape how and when young people access these platforms, with potential downstream effects on both their exposure to and protection from drug-related risks (See: Nash [124] Nash and Felton [125] for a more general discussion of the potential unintended exclusion of children from social media). These developments underscore the need for flexible, future-oriented strategies in platform governance and youth-focused harm reduction.

Furthermore, given the dynamic and evolving nature of online drug markets, it is increasingly important to understand and assess the ways in which licit and illicit drug sales impact emerging digital spaces and platforms, such as the Metaverse [126, 127]. Will the features of these ‘spaces’ used by drug sellers in a way that mirrors those used on social media platforms? While this question falls outside the scope of the current research, it underscores a crucial issue. The field of online drug research must explore how to leverage existing digital spaces on social media to protect and empower young people while preventing the use of these platforms for drug market expansion. As digital spaces evolve and grow, it is increasingly essential to anticipate potential avenues for misuse, and enable the design of protective measures prior to these platforms reaching large-scale adoption [128, 129].

Online safety has emerged as a significant area of academic research and a prominent topic in public discourse and regulatory frameworks, as evidenced by the introduction of legislation in the UK, such as the Online Safety Act, as well as the Kids Online Safety Act in the US [2, 3]. While research on internet and social media-facilitated drug markets has been essential in shedding light on this relatively unexplored area, we argue that it is time to adopt an online safety approach to understanding these markets. This perspective should incorporate technological features and methods relevant to the field, including machine learning techniques, algorithmic recommendations, and digital literacy, thereby reconciling both *drug policy* and *online safety* approaches.

Collaborating with social media companies is, of course, easier said than done. Significant challenges exist, particularly regarding limited accessibility to data [130, 131], especially when it comes to content that violates policies, such as drug-related material. As [132] highlight, automated content moderation can also inadvertently restrict access to harm reduction content, further complicating research and intervention efforts in this space. While there is still a long way to go, taking small steps toward this goal will represent an important initial move in developing a comprehensive approach to online drug safety.

## Limitations

While the survey was distributed through the networks of two drug education charities across the UK, our sample is one of convenience and not representative of the broader UK population. We were unable to gather responses from schools in Northern Ireland and Wales, and most participating schools were state schools. Additionally, 292 responses were excluded due to poor data quality or non-compliance with the ethical consent form. Researching sensitive topics such as drug use among minors requires the highest ethical standards and safeguarding measures, which can limit data availability [133, 134]. The study also relies on self-reported measures, which are prone to social desirability bias and may result in under- or overestimation of behaviours and attitudes, especially in a school setting and among young respondents [135–137]. As a correlational study, we recognise that many confounding and mediating factors, not accounted for here, could influence the relationship between exposure to illicit drugs on social media and young people’s risk perceptions and attitudes. While our findings provide an important initial insight into potential associations between drug exposure on social media and youth attitudes, they require replication. It is also worth noting that nitrous oxide was classified as a Class C drug in the UK in November 2023, after being made illegal for recreational use under the Misuse of Drugs Act [138]. As our survey was conducted between October 2023 and May 2024, some responses may predate this legislative change and therefore may not reflect young people’s current perceptions or behaviours regarding nitrous oxide.

## Conclusions and future directions

This research had two main objectives. First, to understand what school student’s experiences around drugs and social media were, and second, to establish if exposure to illicit drug ads had an influence on their attitudes and behaviours. Young people’s experiences around drugs on social media are varied but common: the presence of drugs on social media and messaging platforms is a routine aspect of their digital lives. We find that seeing illicit drug advertisements is associated with lower risk perceptions of drug ads and a higher tendency to find such content amusing, show interest in it, and to feel unconcerned about the sale of drugs on social media.

We present three key takeaways from our results and their implications for future research. First, platforms matter. They appear to shape users’ exposure to drug-related content, though our findings are situated within the UK context and may not reflect platform dynamics elsewhere. Differences in exposure across platforms can be attributed to the platform’s design, usage patterns among young people, or other underlying factors such as its content moderation processes. In our data, Snapchat and TikTok stood out in terms of the gap between exposure to illicit drug content and harm reduction advice, patterns that merit further investigation in both UK and non-UK settings. Furthermore, future research should extend beyond the traditional and commonly studied platforms, exploring a wider range of platforms, potentially including augmented reality and virtual reality, to understand how illicit drugs may be portrayed or advertised in these spaces. Second, we cannot assert that the presence of drugs on social media and messaging platforms leads to the normalisation or desensitisation of drug use. While the evidence may suggest a relationship, it is crucial to exercise caution in making such claims due to methodological limitations. Other factors may be influencing these dynamics, particularly given the myriad variables associated with digital technology use [139]. Replicating these findings and further disentangling the relationships between key control variables such as social media use, gender, age and outcome variables is a valuable next step. This should be complemented by the inclusion of additional confounding variables and the use of controlled or experimental settings for more rigorous analysis. It will also be important to consider how shifts in drug legislation (e.g. the legalisation of certain substances) and age-based restrictions on social media access may reshape young people’s digital environments and exposure to drug-related risks, presenting new questions for researchers, regulators, and platforms alike. Finally, there is a need to reconsider the framing of research on drugs and social media. While online drug market research has provided invaluable insights, integrating an online safety approach and collaborating with key stakeholders could help shape the field towards outcome-oriented solutions.

## Supplementary Information

Supplementary file 1.

## Data Availability

The datasets generated and analysed during the current study are not publicly available due to ethics agreements but are available from the corresponding author on reasonable request.

## Appendix

See Tables 4, 5, 6, 7, 8, 9, and Fig 7.

**Table 4:** Predictors of risk perceptions of illicit drug ads on social media

Independent variables	Perceived ease of buying drugs on SM (N = 778)		Perceived quality of drugs on SM (N = 362)		Perceived risk of getting caught (N = 573)	
	$\beta$ (SE)	OR	$\beta$ (SE)	OR	$\beta$ (SE)	OR
Having seen drug ads on SM	**1.16****** (0.17)	3.19	**1.00****** (0.22)	2.71	**− 0.70****** (0.18)	0.50
Gender (male)	− 0.18 (0.16)	0.84	0.16 (0.21)	1.17	− 0.26 (0.16)	0.77
Age	0.09 (0.13)	1.10	− 0.09 (0.17)	0.92	− 0.01 (0.13)	1.01
School year (Y11)^a^	− 0.04 (0.26)	0.96	− 0.11 (0.35)	0.82	**− 0.66**** (0.27)	0.52
School year (Y12)	− 0.04 (0.33)	0.96	0.01 (0.41)	1.01	− 0.14 (0.34)	0.87
School year (Y13)	− 0.28 (0.49)	0.76	− 0.19 (0.61)	0.83	− 0.28 (0.50)	0.75
School year (Y9)	0.25 (0.26)	1.29	− 0.34 (0.31)	0.71	0.44 (0.25)	1.55
Scotland	**0.56*** **(0.17)	1.74	0.24 (0.21)	1.27	0.29 (0.17)	1.34
Social media use (less than once a day)^b^	− 0.56 (0.17)	0.57	− 0.20 (0.21)	0.82	− 0.05 (0.17)	0.95
Social media use (once a day)	**− 1.54**** (0.51)	0.21	1.20 (0.66)	3.33	− 0.18 (0.57)	0.84
McFadden’s R2	0.064		0.038		0.037	
Residual deviance	1436.131		875.6944		1225.98	
AIC	1464.131		903.6944		1251.98	

^a^The reference category is ‘Year 10’

^b^The reference category is ‘Hourly social media use’

*p* ≤ 0.05*, *p* ≤ 0.01**, *p* ≤ 0.001***, *p* ≤ 0.0001****

**Table 5:** Predictors of how young people feel around illicit drug ads on social media

a						
Independent variables	Feeling unsafe online (N = 559)		Feeling uncomfortable (N = 582)		Feeling distressed (N = 541)	
	$\beta$ (SE)	OR	$\beta$ (SE)	OR	$\beta$ (SE)	OR
Having seen drug ads on SM	− **1.02********** (0.18)	0.36	− **1.08********** (0.18)	0.34	− **0.81********** (0.19)	0.44
Gender (male)	− **0.66********** (0.16)	0.52	− **0.67*********** (0.16)	0.51	− **0.65********** (0.17)	0.52
Age	− 0.01 (0.14)	0.99	− 0.06 (0.14)	1.94	− 0.07 (0.14)	0.93
School year (Y11)	− 0.21 (0.26)	0.81	0.25 (0.26)	1.28	− 0.08 (0.27)	0.93
School year (Y12)	0.02 (0.33)	1.02	0.33 0.33)	1.38	− 0.20 (0.35)	0.81
School year (Y13)	− 0.08 0.51)	0.92	0.62 (0.49)	1.87	0.26 (0.52)	1.29
School year (Y9)	**0.58******** (0.25)	1.78	**0.68******** (0.25)	1.96	0.31 0.26)	1.37
Scotland	− **0.38******* (0.17)	0.68	− **0.39******** (0.17)	0.67	− 0.10 0.18)	0.90
Social media use (less than once a day)	0.27 (0.17)	1.31	**0.42******** (0.17)	1.52	0.13 (0.18)	1.14
Social media use (once a day)	0.68 0.48)	1.98	**0.95******* (0.48)	2.59	− 0.02 (0.50)	0.98
McFadden’s R2	0.059		0.064		0.040	
Residual deviance	1304.917		1364.529		1183.859	
AIC	1332.917		1392.529		1211.859	

b						
Independent variables	Feeling interested (N = 583)		Feeling amused (N = 554)		Not feeling concerned (N = 553)	
	$\beta$ (SE)	OR	$\beta$ (SE)	OR	$\beta$ (SE)	OR
Having seen drug ads on SM	**1.02********** (0.18)	2.77	**0.88********** (0.18)	2.42	**0.48******** (0.18)	1.61
Gender (male)	0.00 (0.16)	1.00	− 0.11 (0.16)	0.90	0.22 (0.16)	1.25
Age	**0.28*** 0.13)	1.32	**0.27******* (0.13)	1.32	0.00 (0.14)	1.00
School year (Y11)	− 0.19 0.26)	0.82	0.00 (0.26)	1.00	0.10 (0.26)	1.11
School year (Y12)	− **0.64******* 0.33)	0.53	− 0.30 (0.32)	0.74	0.37 (0.33)	1.45
School year (Y13)	− 0.77 0.49)	0.46	− 0.64 (0.46)	0.53	0.03 (0.50)	1.03
School year (Y9)	− 0.12 (0.25)	0.89	− 0.32 (0.25)	0.73	0.01 (0.26)	1.01
Scotland	− 0.11 (0.17)	0.90	0.12 (0.17)	1.12	0.01 (0.17)	1.01
Social media use (less than once a day)	0.53 (0.17)	0.59	− 0.21 (0.17)	0.81	− **0.36******* (0.17)	0.70
Social media use (once a day)	− 0.91 (0.50)	0.40	− 0.73 (0.46)	0.48	00.2 (0.45)	1.02
McFadden’s R2	0.055		0.043		0.016	
Residual deviance	1269.589		1380.981		1394.898	
AIC	1297.589		1408.981		1422.898	

*p* ≤ 0.05*, *p* ≤ 0.01**, *p* ≤ 0.001***, *p* ≤ 0.0001****

**Table 6:** Predictors of purchasing drugs on social media

Independent variables	Having bought drugs on SM (N = 695)	
	$\beta$(SE)	OR
Having seen drug ads on SM	**2.86********** (0.34)	17.39
Gender (male)	− **0.54******* (0.27)	0.58
Age	0.34 (0.22)	1.41
School year (Y11)	− 0.41 (0.44)	0.66
School year (Y12)	− 0.69 (0.59)	0.50
School year (Y13)	− 0.69 (0.78)	0.50
School year (Y9)	0.04 (0.49)	1.04
Scotland	0.07 (0.28)	1.07
Social media use (less than once a day)	− **0.95********* (0.29)	0.39
Social media use (once a day)	− 0.54 (1.13)	0.58
McFadden’s R2	0.287	
Residual deviance	379.16	
AIC	401.16	

*p* ≤ 0.05*, *p* ≤ 0.01**, *p* ≤ 0.001***, *p* ≤ 0.0001****

**Table 7:** Predictors of thinking illicit drug ads on social media are a problem

Independent variables	Perceiving drug ads on SM as a problem (N = 649)	
	$\beta$ (SE)	OR
Having seen drug ads on SM	**0.34******* (0.16)	1.41
Gender (male)	**0.32******* (0.15)	1.38
Age	0.04 (0.12)	1.04
School year (Y11)	− 0.10 (0.23)	0.90
School year (Y12)	− 0.28 (0.30)	0.75
School year (Y13)	− 0.30 (0.43)	0.74
School year (Y9)	− 0.10 (0.24)	0.90
Scotland	− 0.11 (0.16)	0.90
Social media use (less than once a day)	**0.44******** (0.16)	0.64
Social media use (once a day)	− 0.66 (0.52)	0.52
McFadden’s R2	0.016	
Residual deviance	1367.299	
AIC	1391.299	

*p* ≤ 0.05*, *p* ≤ 0.01**, *p* ≤ 0.001***, *p* ≤ 0.0001****

**Table 8:** Predictors of barriers to reporting illicit drug ads on social media

Independent variables	Not reporting because they can’t be bothered (N = 778)		Not reporting because it doesn’t make a difference (N = 778)		Not reporting because they don’t want to be a snitch (N = 778)	
	$\beta$ (SE)	OR	$\beta$ (SE)	OR	$\beta$ (SE)	OR
Having seen drug ads on SM	0.07 (0.16)	1.00	**0.58********* (0.17)	1.78	**0.60********* (0.19)	2
Gender (male)	0.21 (0.15)	1.24	− 0.27 (0.16)	0.76	− **0.38******* (0.18)	0.69
Age	0.13 (0.11)	1.14	− 0.07 (0.12)	0.94	− 0.10 (0.14)	1.10
School year (Y11)	− 0.08 (0.24)	0.93	0.29 (0.26)	1.34	0.19 (0.28)	1.21
School year (Y12)	− 0.28 (0.30)	0.76	0.59 (0.31)	1.81	− 0.40 (0.37)	0.67
School year (Y13)	− **1.02******* (0.44)	0.36	**1.00******* (0.46)	2.73	− 1.05 (0.57)	0.35
School year (Y9)	− 0.24 (0.23)	0.79	− 0.14 (0.26)	0.87	0.34 (0.27)	1.40
Scotland	− 0.13 (0.15)	0.88	− **0.44******** (0.17)	0.65	0.25 (0.19)	1.28
Social media use (less than once a day)	− 0.07 (0.16)	0.93	0.22 (0.17)	1.25	− 0.06 (0.19)	0.94
Social media use (once a day)	− 0.61 (0.48)	0.55	− 0.28 (0.53)	0.75	0.20 (0.54)	1.22
McFadden’s R2	0.014		0.038		0.037	
Residual deviance	1042.4		952.15		787	
AIC	1064.4		974.15		809	

*p* ≤ 0.05*, *p* ≤ 0.01**, *p* ≤ 0.001***, *p* ≤ 0.0001****

**Table 9:** Predictors of perceived responsibility of different actors regarding illicit drug ads on social media

a				
Independent variables	Responsibility of social media companies (N = 695)		Responsibility of the police (N = 695)	
	$\beta$ (SE)	OR	$\beta$ (SE)	OR
Having seen drug ads on SM	− **0.38******* (0.19)	0.69	− **0.39******* (0.20)	0.68
Gender (male)	− 0.28 (0.18)	0.75	− 0.18 (0.18)	0.84
Age	0.22 (0.13)	1.25	0.19 (0.13)	1.21
School year (Y11)	0.18 (0.29)	1.20	**1.03********* (0.31)	2.81
School year (Y12)	− 0.37 (0.34)	0.69	0.06 (0.34)	1.06
School year (Y13)	− 0.59 (0.50)	0.56	− 0.36 (0.50)	0.70
School year (Y9)	− 0.30 (0.26)	0.74	− 0.13 (0.26)	0.88
Scotland	− 0.01 (0.18)	0.99	0.21 (0.19)	1.24
Social media use (less than once a day)	**0.82********** (0.19)	2.27	**0.65********* (0.19)	1.91
Social media use (once a day)	0.70 (0.58)	2.02	− 0.33 (0.49)	0.72
McFadden’s R2	0.049		0.058	
Residual deviance	779.31		771.74	
AIC	801.31		793.74	

b				
Independent variables	Responsibility of the government (N = 695)		Responsibility of schools (N = 695)	
	$\beta$ (SE)	OR	$\beta$ (SE)	OR
Having seen drug ads on SM	− **0.43******* (0.19)	0.65	− 0.27 (0.18)	0.76
Gender (male)	− 0.12 (0.18)	0.89	− **0.36******* (0.16)	0.70
Age	0.10 (0.13)	1.10	0.19 (0.12)	1.21
School year (Y11)	**0.70******* (0.30)	2.02	0.26 (0.25)	1.30
School year (Y12)	− 0.11 (0.34)	0.90	− 0.13 (0.31)	0.87
School year (Y13)	− 0.14 (0.49)	0.87	− 0.64 (0.45)	0.53
School year (Y9)	− 0.29 (0.26)	0.75	− 0.33 (0.24)	0.72
Scotland	0.02 (0.18)	1.02	**0.37******* (0.17)	1.44
Social media use (less than once a day)	**0.66********* (0.19)	1.93	**0.38******* (0.17)	1.47
Social media use (once a day)	− 0.35 (0.49)	0.70	− 0.33 (0.48)	0.72
McFadden’s R2	0.044		0.032	
Residual deviance	788.56		931.88	
AIC	810.56		953.88	

*p* ≤ 0.05*, *p* ≤ 0.01**, *p* ≤ 0.001***, *p* ≤ 0.0001****

## Footnotes

1 https://talkabouttrust.org/

2 The total does not equal to 1151 as there were 32 NA answers for this question.

3 Percentages rounded and may not sum to exactly 100.

4 While opioids and benzodiazepines are pharmacologically distinct, they are both central nervous system depressants and were grouped together for the purpose of this analysis.
