# Supplementary material for: Platforms, risk perceptions, and reporting: the impact of illicit drug advertisements on social media among UK secondary students
Source: Harm Reduct J. 2025 Oct 3;22:154. doi: 10.1186/s12954-025-01299-5 (PMC12495684; doi:10.1186/s12954-025-01299-5)
Supplement: Supplementary file 1 [file 12954_2025_1299_MOESM1_ESM.pdf]

UCL

Consent form

CONSENT FORM FOR PARTICIPANTS

Thank you for taking part in this research. Before taking the survey, you need to complete this consent form. You should have read the Information Sheet and listened to an explanation about the research.

📚 **Title of Study**: Drugs on Social Media and Young People.
🔎 **Department:** UCL Jill Dando Institute of Security and Crime Science
📩 **Contact**: uclsocialmediaproject@gmail.com

This study has been approved by the UCL Research Ethics Committee: Project ID number: 23897/001

If you have any questions about from the information sheet or explanation already given to you, please ask your teacher or supervisor before you decide whether to join in.

Please tick yes or no according to the following statements

	Yes✅	No❌
I have read and understood the information sheet.	☐	☐
I understand that the answers I provide in the survey will be used for the purposes explained.	☐	☐
I understand that personal information will remain confidential and that I cannot be identified unless someone might be in immediate danger, in which case we have a duty to inform the school of this.	☐	☐

	Yes✅	No❌
I understand that I will be able to withdraw my data up to 6 months after taking the survey.	☐	☐
I understand the support that will be available to me if I become distressed during the survey.	☐	☐
I understand that the data will not be made available to any commercial organisations.	☐	☐
I consent and agree to take part in this study.	☐	☐

⚠️ In the next questions, you will be asked for your name and school name **only** for safeguarding purposes. Your name and your answers **will only be linked in case** there are reasons for us to believe you are in danger. ⚠️

Circumstances that would make us believe this are:
⛔ if you say you are having negative thoughts and want to self-harm
⛔ if you are being threatened
⛔ if you feel in danger
⛔ if you are struggling with addiction

What is your name? **This information will not be kept, analysed nor published in the results.**

What is the name of your school? **This information will not be kept, analysed nor published in the results.**

Introduction

This survey is 🔒 **completely confidential** 🔒 so please be as honest as you can.

The aim is to find out more about the experiences of teenagers using social media, and how seeing drugs on such platforms might affect them. We want to make sure young people are safe around drugs, both online and offline. The survey will help us understand your experiences better, and figure out whether we can do anything to make things safer.

Here, drugs are considered to be any illicit/illegal drug, including controlled prescription drugs, such as Valium and Tramadol.

T1.1 [Part 1]

Did you know? 73% of young people said they reported illegal and hamful posts on social media.

Did you spot the typo in the message that you just saw? 😉 Which word was it?

T1.2 [Part 1]

Did you know? 23% of young people said they reported illegal and hamful posts on social media.

Did you spot the typo in the message that you just saw? 😉 Which word was it?

T2 [Part 1]

Did you know? Reporting a post or user for illegal or harmful content is totally anonymous. They will not know who has made the repot.

Did you spot the typo in the message that you just saw?😉 Which word was it?

T3 [Part 1]

Did you know? Reporting illegal or harmful content on social media makes a difference. This helps platforms improve algorithms to beter detect illegal and harmful content.

Did you spot the typo in the message that you just saw? 😉 Which word was it?

Control [Part 1]

Did you know? Sixdegrees.com was considered the “first” social media platfom in history and was founded in 1997.

Did you spot the typo in the message that you just saw?😉 Which word was it?

[ ]

Demographics

How old are you?

- ○ 13
- ○ 14
- ○ 15
- ○ 16
- ○ 17
- ○ 18

In which school year are you?

- ○ Year 9
- ○ Year 10
- ○ Year 11
- ○ Year 12
- ○ Year 13

What is your gender?

- ○ Male
- ○ Female
- ○ Non-binary / third gender
- ○ Prefer not to say

What type of school do you go to?

- ○ State school
- ○ Faith school
- ○ Grammar school
- ○ Independent school
- ○ FE/Sixth form college
- ○ [ ] Other (please specify)

In which county do you live?

[ ]

Social media use

Do you use social media sites or apps? This may include looking at social media apps, uploading or sharing videos or photos online or sharing links to websites or online articles.

- ○ Yes
- ○ No

How often do you use social media? **Please select only those that apply.**

	Every hour	Several times a day	Once a day	Several times a week	Once a week	Less than once a week	Never
Facebook	☐	☐	☐	☐	☐	☐	☐
Instagram	☐	☐	☐	☐	☐	☐	☐
Snapchat	☐	☐	☐	☐	☐	☐	☐
TikTok	☐	☐	☐	☐	☐	☐	☐
Tumblr	☐	☐	☐	☐	☐	☐	☐
X (formely known as Twitter)	☐	☐	☐	☐	☐	☐	☐
YouTube	☐	☐	☐	☐	☐	☐	☐
Discord	☐	☐	☐	☐	☐	☐	☐
Pinterest	☐	☐	☐	☐	☐	☐	☐
Other (please specify) [ ]	☐	☐	☐	☐	☐	☐	☐
BeReal	☐	☐	☐	☐	☐	☐	☐
Craigslist	☐	☐	☐	☐	☐	☐	☐
WhatsApp	☐	☐	☐	☐	☐	☐	☐
Signal	☐	☐	☐	☐	☐	☐	☐
Telegram	☐	☐	☐	☐	☐	☐	☐
Wickr	☐	☐	☐	☐	☐	☐	☐
Depop	☐	☐	☐	☐	☐	☐	☐
Vinted	☐	☐	☐	☐	☐	☐	☐

Drugs and drug ads on social media

Have you ever seen content about drugs in general on social media? This may include videos or posts about other people consuming drugs, doing a challenge, news...

- ○ Yes
- ○ No
- ○ Prefer not to say

What type of content was this and on which platform was it? **Please select only those that apply.**

	Funny content/memes	Educational content	Other people consuming	Challenges or Tricks	Legal advertisements for drugs	Ne
Facebook	☐	☐	☐	☐	☐	☐
Instagram	☐	☐	☐	☐	☐	☐
Snapchat	☐	☐	☐	☐	☐	☐
TikTok	☐	☐	☐	☐	☐	☐
Tumblr	☐	☐	☐	☐	☐	☐
X (formely known as Twitter)	☐	☐	☐	☐	☐	☐
YouTube	☐	☐	☐	☐	☐	☐
Discord	☐	☐	☐	☐	☐	☐
Pinterest	☐	☐	☐	☐	☐	☐
Other (please specify) ____	☐	☐	☐	☐	☐	☐
BeReal	☐	☐	☐	☐	☐	☐
Craigslist	☐	☐	☐	☐	☐	☐
WhatsApp	☐	☐	☐	☐	☐	☐
Telegram	☐	☐	☐	☐	☐	☐
Signal	☐	☐	☐	☐	☐	☐
Wickr	☐	☐	☐	☐	☐	☐
Depop	☐	☐	☐	☐	☐	☐
Vinted	☐	☐	☐	☐	☐	☐

What type of drugs were shown? **Please select all that apply.**

- ☐ Steroids
- ☐ Don't know
- ☐ Nitrous oxide/Laughing gas/NOS or balloons
- ☐ Cocaine/Crack cocaine

- ☐ Mephredone
- ☐ LSD/Acid
- ☐ ______ Other (please specify)
- ☐ Valium
- ☐ MDMA/Ectasy
- ☐ Cannabis edibles, including cannabis gummies
- ☐ Codeine or cough syrup mix/Lean
- ☐ Cannabis/THC vape liquids
- ☐ Tramadol
- ☐ Heroin
- ☐ Xanax
- ☐ Amphetamines/Speed/Crystal meth
- ☐ Cannabis/weed, including cannabis-based products such as concentrates, oils and hash
- ☐ Synthetic cannabinoids/'Spice'/'Black Mamba'
- ☐ Ketamine
- ☐ Magic mushrooms

Have you or anyone you know ever seen illegal drugs being advertised for sale on social media?

- ○ Yes
- ○ No
- ○ Prefer not to say

Was this a result of searching for drugs or did drug adverts appear without searching?

- ○ A result of searching for drugs
- ○ A result of drugs being advertised for sale without searching
- ○ Both

Where and how often have you seen the posts advertising drugs for sale? **Please select only those that apply.**

	Several times a day	Once a day	Several times a week	Once a week	Several times a month	Less often
Facebook	○	○	○	○	○	○
Instagram	○	○	○	○	○	○
Snapchat	○	○	○	○	○	○

	Several times a day	Once a day	Several times a week	Once a week	Several times a month	Less often
TikTok	○	○	○	○	○	○
Tumblr	○	○	○	○	○	○
X (formely known as Twitter)	○	○	○	○	○	○
YouTube	○	○	○	○	○	○
Discord	○	○	○	○	○	○
Pinterest	○	○	○	○	○	○
Other (please specify) ____	○	○	○	○	○	○
BeReal	○	○	○	○	○	○
Craigslist	○	○	○	○	○	○
WhatsApp	○	○	○	○	○	○
Signal	○	○	○	○	○	○
Telegram	○	○	○	○	○	○
Wickr	○	○	○	○	○	○
Depop	○	○	○	○	○	○
Vinted	○	○	○	○	○	○

What type of drugs were these? **Please select all that apply.**

- ☐ Codeine or cough syrup mix/Lean
- ☐ Amphetamines/Speed/Crystal meth
- ☐ ____ Other (please specify)
- ☐ Steroids
- ☐ LSD/Acid
- ☐ Synthetic cannabinoids/‘Spice’/‘Black Mamba’
- ☐ Cocaine/Crack cocaine
- ☐ Heroin
- ☐ Valium
- ☐ Cannabis edibles, including cannabis gummies
- ☐ Tramadol
- ☐ Mephredone
- ☐ MDMA/Ectasy
- ☐ Magic mushrooms
- ☐ Xanax
- ☐ Ketamine
- ☐ Cannabis/THC vape liquids

- ☐ Don't know
- ☐ Cannabis/weed, including cannabis-based products such as concentrates, oils and hash
- ☐ Nitrous oxide/Laughing gas/NOS or balloons

Which of the following age ranges best describes the age of the people you have seen advertising drugs?

- ☐ 12 and under
- ☐ 13 to 17
- ☐ 18 to 24
- ☐ 25 to 34
- ☐ 35 to 44
- ☐ 45 and above

Drug ads and behaviours/attitudes

In your opinion, how easy do you think it is to buy illegal drugs on social media?

- ○ Very easy
- ○ Easy
- ○ Not easier or harder
- ○ Difficult
- ○ Very difficult
- ○ Don't know

In your opinion, do you think the drugs being sold on social media are of higher quality than those sold on the street?

- ○ Strongly agree
- ○ Agree
- ○ Neither agree or disagree
- ○ Disagree
- ○ Strongly disagree
- ○ Don't know

In your opinion, how likely is it for people to get caught when buying or selling drugs on social media?

- ○ Extremely unlikely

- Unlikely
- Likely
- Extremely likely
- Don't know

Looking at the following statements, please select all that apply. "*Seeing illegal drugs advertised for sale on social media...*"

	Strongly disagree	Disagree	Agree	Strongly agree	Don't know
Has made me or someone I know feel less safe online	O	O	O	O	O
Has made me or someone I know feel uncomfortable	O	O	O	O	O
Has made me or someone I know feel distressed	O	O	O	O	O
Has made me or someone I know interested in the content	O	O	O	O	O
Has made me or someone I know laugh	O	O	O	O	O
Does not concern me or the person I know	O	O	O	O	O

Looking at the following statements, please select all that apply. "*Seeing illegal drugs advertised for sale on social media...*"

	Strongly disagree	Disagree	Agree	Strongly agree	Don't know
Would make me or someone I know feel less safe online	O	O	O	O	O
Would make me or someone I know feel uncomfortable	O	O	O	O	O
Would make me or someone I know feel distressed	O	O	O	O	O
Would make me or someone I know interested in the content	O	O	O	O	O
Would make me or someone I know laugh	O	O	O	O	O

	Strongly disagree	Disagree	Agree	Strongly agree	Don't know
Would not concern me or the person I know	○	○	○	○	○

Drug buying characteristics

Have you or someone you know ever bought any illegal drugs through social media?

- ○ Yes
- ○ No
- ○ Prefer not to say

Which apps were used to organise delivery? **Please select all that apply.**

- ☐ Instagram
- ☐ WhatsApp
- ☐ Telegram
- ☐ ________ Other (please specify)
- ☐ Wickr
- ☐ Tumblr
- ☐ Don't know
- ☐ Facebook
- ☐ X (formely known as Twitter)
- ☐ Snapchat
- ☐ TikTok
- ☐ Text message
- ☐ YouTube

Which payment methods were used? **Please select all that apply.**

- ☐ Cash on delivery
- ☐ Bank transfer (including online banks like Revolut or Monzo)
- ☐ Cryptocurrency transfer
- ☐ PayPal
- ☐ Cash App
- ☐ MoneyGram
- ☐ Online gift card
- ☐ ________ Other (please specify)
- ☐ Don't know

How were the drugs delivered? **Please select all that apply.**

- ☐ By a friend
- ☐ By the dealer at an arranged place
- ☐ By the dealer to the home
- ☐ Through the post
- ☐ [ ] Other (please specify)
- ☐ Don't know

Reporting practices and prevention

Have you ever reported/told to the following people about the advertising of illegal drugs on social media?

	Yes	No	Prefer not to say
Social media platform	○	○	○
The police	○	○	○
A parent or guardian	○	○	○
School staff member	○	○	○

How often have you reported this?

	Every time	Most times	Occasionally	Rarely
» Social media platform	○	○	○	○
» The police	○	○	○	○
» A parent or guardian	○	○	○	○
» School staff member	○	○	○	○

How often have you seen the same account advertising drugs for sale ***after*** you have reported it on social media?

- ○ Frequently
- ○ Occasionally
- ○ Does not change, I see it the same amount of times
- ○ Rarely
- ○ Never

If you have seen illegal drugs advertised and not reported it, what stopped you? **Please**

select all that apply

- ☐ I don't think it would make any difference
- ☐ I didn't realise I could report them
- ☐ I'm worried I would get in trouble too as friends bought drugs off them
- ☐ I'm not sure how to report them
- ☐ Prefer not to say
- ☐ The person advertising is a friend or a mutual friend
- ☐ I'm worried I would get in trouble too as I've chosen to follow this account
- ☐ I don't want to get the person shut down/account banned because the adverts are funny/interesting
- ☐ ________ Other (please specify)
- ☐ I don't want to be a snitch
- ☐ The person advertising discouraged me from doing so
- ☐ I don't think they are doing any harm
- ☐ I can't be bothered
- ☐ I would worry the person I reported finding out
- ☐ I don't want to get the person shut down/account banned because they are where people source drugs

Imagine you have seen illegal drugs advertised on social media. What would stop you from reporting illicit drugs advertisements on social media? **Please select all that apply**

- ☐ I wouldn't want to get the person shut down/account banned because they are where people source drugs
- ☐ I would worry the person I reported finding out
- ☐ The person advertising is a friend or a mutual friend
- ☐ ________ Other (please specify)
- ☐ I didn't realise I could report them
- ☐ I wouldn't be sure how to report them
- ☐ I woudn't want to be a snitch
- ☐ I would think it wouldn't make any difference
- ☐ I wouldn't want to get the person shut down/account banned because the adverts are funny/interesting
- ☐ I would be worried I would get in trouble too as friends bought drugs off them
- ☐ I wouldn't think they are doing any harm
- ☐ Prefer not to say
- ☐ I would be worried I would get in trouble too as I've chosen to follow this account
- ☐ The person advertising may have discouraged me from doing so
- ☐ I wouldn't be bothered

Is there anything that would encourage you to report illegal drug advertisements on social media? If yes, what would this be?

Do you think illegal drugs being advertised and sold on social media is a problem that needs to be solved? Why?

- No , because...
- Yes, because...
- Maybe, because...

In your opinion, to what extent should the following people be tasked to stop drugs being advertised on social media?

	Not responsible	Somewhat responsible	Responsible	Prefer not to say
Social media companies	○	○	○	○
The police	○	○	○	○
The goverment	○	○	○	○
Schools	○	○	○	○
Other (please specify)	○	○	○	○

Did you know that some accounts advertising illegal drugs on social media are scams? (*eg. Asking for money but not sending any drugs*)

- Yes
- No
- Prefer not to say

Have you or anyone you know seen any accounts or content promoting susbtance safety advice on your social media feed? (*eg. A campaign informing of the risks of drugs on mental health*)

- Yes
- No
- Prefer not to say

Where did you see substance safety advice and how frequently did you see it? **Please select only those that apply.**

	Several times a day	Once a day	Several times a week	Once a week	Several times a month	Less often
Facebook	○	○	○	○	○	○
Instagram	○	○	○	○	○	○
Snapchat	○	○	○	○	○	○
TikTok	○	○	○	○	○	○
Tumblr	○	○	○	○	○	○
X (formely known as Twitter)	○	○	○	○	○	○
YouTube	○	○	○	○	○	○
Discord	○	○	○	○	○	○
Pinterest	○	○	○	○	○	○
Other (please specify) [ ]	○	○	○	○	○	○
BeReal	○	○	○	○	○	○
Craigslist	○	○	○	○	○	○
WhatsApp	○	○	○	○	○	○
Telegram	○	○	○	○	○	○
Signal	○	○	○	○	○	○
Wickr	○	○	○	○	○	○
Depop	○	○	○	○	○	○
Vinted	○	○	○	○	○	○

Free text box

Is there anything else you would like to tell us?

[ ]

Experiment [Part 2, Question]

TASK TIME! 💭 Imagine you are on your phone, scrolling through your social media feed... 🥱
